# Decipher ‘Em All: A Profiling Study on the Effects of Acyl Groups in *O*-Acyl-ω-hydroxy Fatty Acids

**DOI:** 10.1021/acs.langmuir.4c02469

**Published:** 2024-10-03

**Authors:** Henrik Stubb, Julia Sevón, Cordula Schlegel, Mira Viljanen, Jukka Moilanen, Tuomo Viitaja, Filip S. Ekholm

**Affiliations:** † Department of Chemistry, University of Helsinki, P.O. Box 55, FI-00014 Helsinki, Finland; ‡ Ophthalmology, University of Helsinki and Helsinki University Hospital, Haartmaninkatu 8, FI-00290 Helsinki, Finland

## Abstract

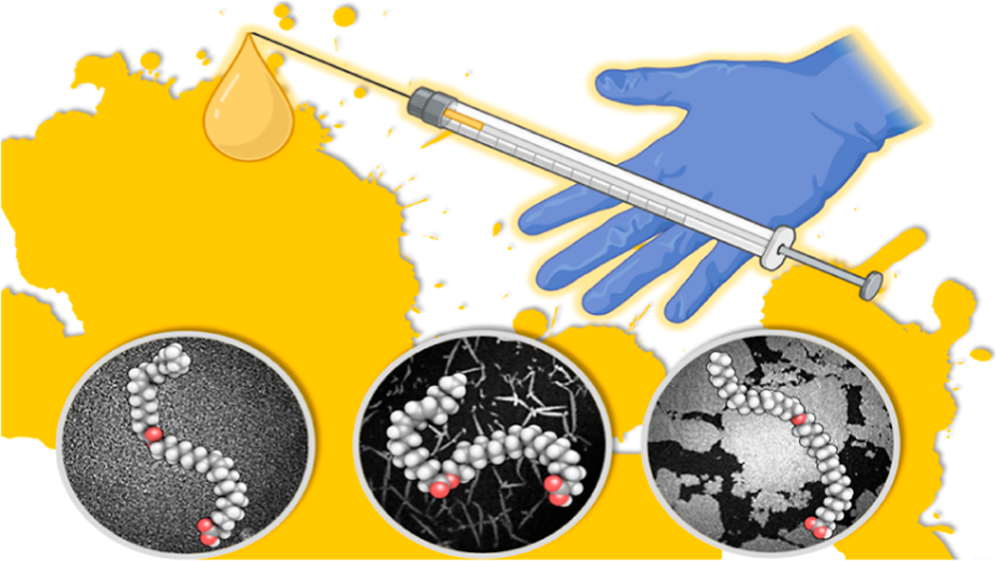

The *O*-acyl-ω-hydroxy fatty acids (OAHFAs) are an intriguing class of surface-active lipids which can be found in the human tear film lipid layer (TFLL). Recent studies have suggested that OAHFAs exist in the polar lipid layer and play a central role in TFLL function. Surprisingly, biophysical profiling studies have only shed light on the properties of OAHFAs bearing an oleate acyl group and insights on species with other acyl groups are scarce. Herein, we seek to address this issue through (1) focusing on the synthesis and characterization of a representative library of OAHFA analogues bearing a palmitate, palmitoleate, stearate, and linoleate acyl group, and (2) performing an in-depth mapping of their biophysical properties. Our results indicate that NMR-spectroscopic techniques can be utilized for rough estimation of the amounts of distinct acyl groups in a sample and more importantly, how the subtle variations in both parent chains and acyl groups influence the core properties of the OAHFAs. We reach the conclusion that the correlation between melting points and film properties is not as clear-cut as previously thought. Nevertheless, grouping of OAHFA species into three separate categories which display distinct behavior seems to be possible utilizing the melting points as a guiding parameter. Altogether, our study suggests that the properties of OAHFAs need to be assessed from a viewpoint which combines both the parent chain and acyl group instead of independent analysis based on either fragment alone.

## Introduction

The tear film lipid layer (TFLL) forms the outermost layer of the human tear film and plays a vital role in maintenance of ocular surface health.^1−3^ On a general level, the TFLL is thought to consist of a thin amphiphilic sublayer, which is in direct contact with the aqueous tear film layer, covered by a thicker nonpolar layer which in turn is in contact with both the amphiphilic sublayer and with air.^4^ One of the key lipid classes in the amphiphilic sublayer is the *O*-acyl-ω-hydroxy fatty acids (OAHFAs). The existence of these species was originally proposed by Nicolaides and Santos in 1985^5^ but proven much later independently by the teams of Butovich et al. in 2009 and Chen et al. in 2010.^6,7^ Although the OAHFAs make up only 1–4% of the total lipid content in the TFLL,^8−12^ recent studies have indicated that their intrinsic properties (surface activity, spreadability, and for selected species, evaporation resistance) may be central to maintaining a functional TFLL on the whole.^13−15^

**Figure 1:**
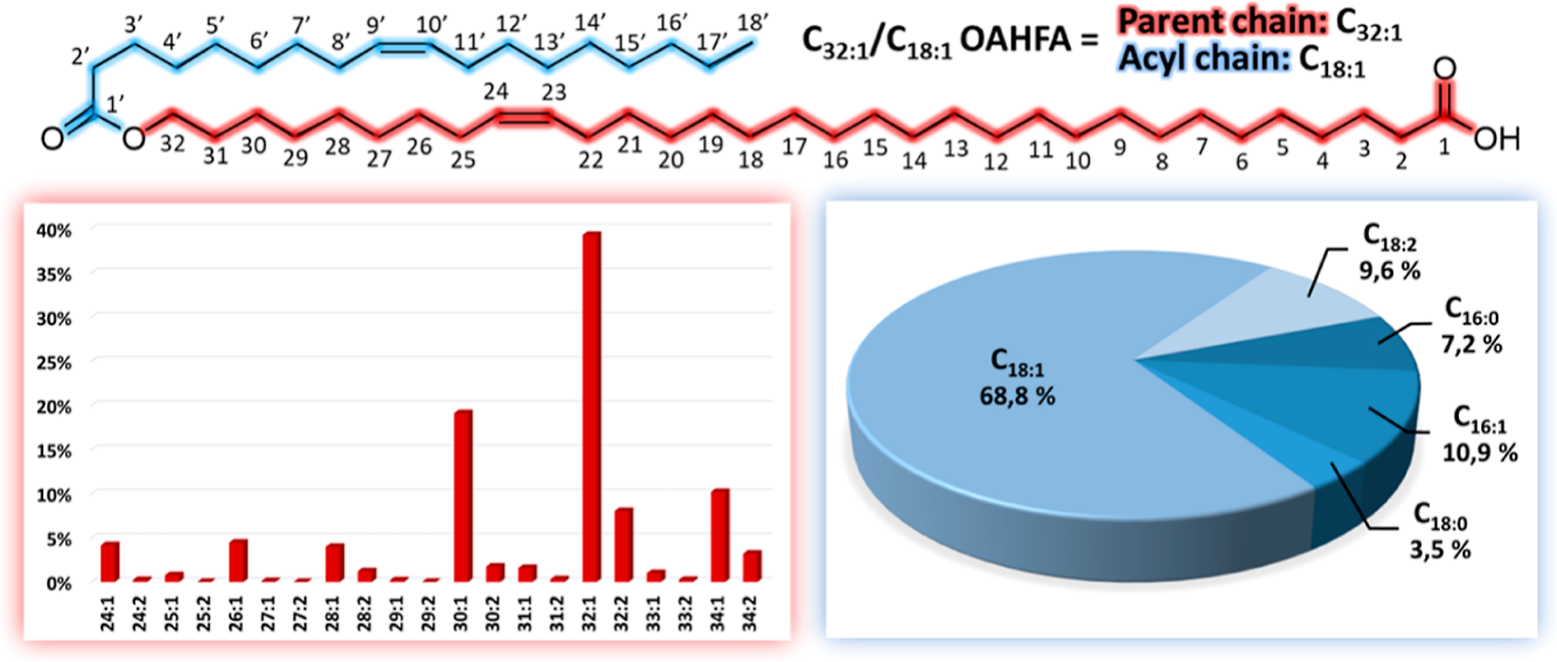
Top: a representative chemical structure of a 32:1/18:1-OAHFA highlighting the correlation between abbreviations used in the text and the chemical structure. Bottom: statistical insights on the distribution of OAHFA species (in mol %) in tear samples based on the data reported by Brown et al.^10^ Bottom left: variations in the parent chain are highlighted. Bottom right: variations in the acyl groups are highlighted.

The TFLL OAHFAs are a diverse set of molecules (see Figure 1) consisting of predominantly even-numbered, monounsaturated parent carbon chains with lengths ranging from C_24_ to C_34_ to which an acyl group is attached at the ω-hydroxy group [the major acyl groups are palmitate (C_16:0_), palmitoleate (C_16:1_), stearate (C_18:0_), oleate (C_18:1_), and linoleate (C_18:2_)].^6,10,11^ The most prominent parent carbon chain is the C_32:1_ and the most common acyl group is the C_18:1_. Thus, it is unsurprising that the previous synthesis and profiling studies in the field have focused on studying naturally occurring/model compounds featuring either of these fragments.^13,15−20^ However, ∼30% of the OAHFAs bear an acyl group distinct from the oleate and insights on the properties of such species are scarce within the TFLL context. This is surprising since it is well-known that structural features such as degree of saturation, chain length, and branching do have a major impact on the biophysical properties displayed by lipid films.^21−24^ To build an overarching understanding on the properties displayed by this intriguing TFLL lipid class, it is important to supplement the previous studies in the field with a dedicated biophysical profiling study focused on the effects of distinct acyl groups. Herein, we undertake this specific task.

## Results and Discussion

During our previous work on the synthesis and biophysical profiling of simplified model compounds,^13^ and shorter/longer OAHFA-species resembling the naturally occurring ones,^17,25^ we noted that the fundamental biophysical profiles of this lipid class can be modeled to a reasonable degree by the use of simplified structural analogues. For example, a C_20:0_/C_18:1_-OAHFA model compound bears a strikingly similar biophysical profile as the weighted average TFLL OAHFA, the C_29:1_/C_18:1_-OAHFA.^15,26^ Therefore, we considered that an assessment platform for evaluating the effects of acyl groups could be successfully established on the C_20:0_-parent carbon chain. This would notably shorten the synthesis routes required to access a representative library of compounds for profiling purposes. Additionally, to assess the other end of the OAHFA-spectrum (modeling shorter chain lengths than the C_29:1_/C_18:1_), we included an additional C_12:0_-parent chain. In summary, we set out to create an OAHFA-library with two variations in the parent chain length (C_20:0_ and C_12:0_) and four variations in the acyl group (C_16:0_, C_16:1_, C_18:0_, C_18:2_). The corresponding oleate (C_18:1_) derivatives have been synthesized and studied earlier^13^ and these results will be treated as a valuable reference throughout the discussion below.

### Synthesis and Structural Characterization of an OAHFA Library

#### Chemical Synthesis

For construction of the OAHFA library, we decided to utilize our previously reported two-step monoacylation-oxidation sequence (Table 1).^13^ The monoacylation was performed as an acid catalyzed Fischer esterification under neat reaction conditions at elevated temperatures (90–100 °C). Typically, sulfuric acid is used as a catalyst.^27^ However, to avoid isomerization of *Z*-alkenes into *E*-alkenes a milder acid catalyst was employed, i.e., 3.5 mol % of sodium hydrogen sulfate. Under these conditions isomerization of the double bond does not occur. While the reactions and purifications are straightforward, the yields are moderate—typically in the range of 30–50% as can be seen from the monoacylated products **1**–**8**. The reaction is however not easily optimized. For example, the reaction temperature needs to be sufficiently high to allow the compounds to react in the liquid state, and increasing the amount of acylating agent above one equivalent is generally accompanied by an increase in the formation of diester species. Thus, further optimization of the reaction conditions was not attempted in the current study. Instead, the monoacylated products **1**–**8** were oxidized to the corresponding OAHFAs utilizing Jones’ reagent (CrO_3_ in H_2_SO_4_). Chromium-based oxidation agents are not ideal due to their toxicity, but they are very efficient and tolerate the other functional groups present in the monoacylated intermediates. Herein, the oxidation proceeded to completion within a few hours at ambient temperature and the OAHFAs **9**–**16** were isolated following purification in yields ranging from 71 to 92%. This gave us access to a representative library featuring two variations in parent chain length and four variations in the acyl group. Importantly, the reactions employed were found to be robust and we did not discover distinct reactivity trends within the substrate scope used. In other words, the reaction efficiency was not affected by chain length or degree of saturation.

**Table 1:**
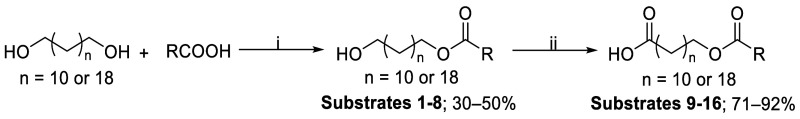

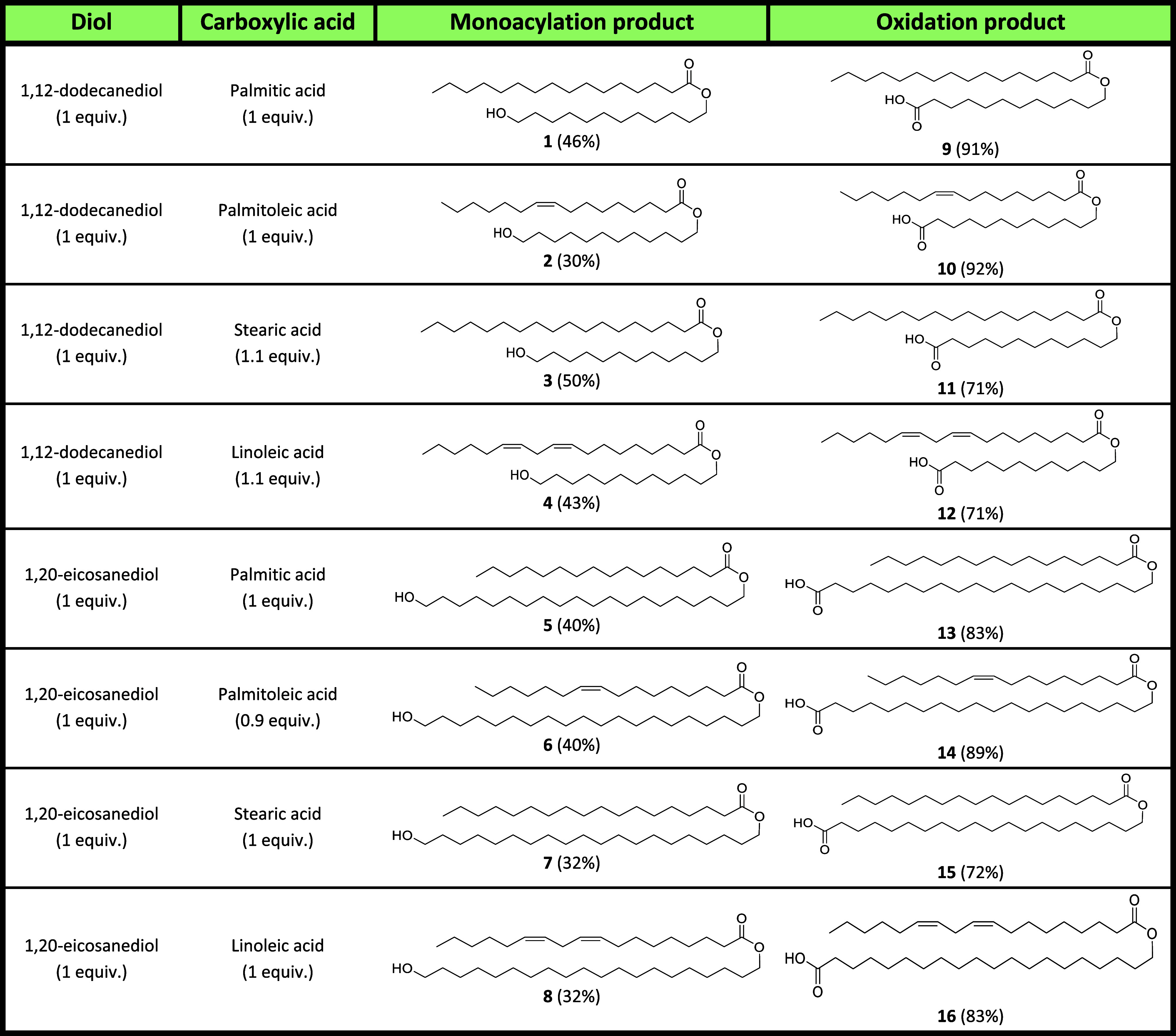
Reaction Conditions and Synthesized Substrates Given in the Form of a General Reaction Scheme and a Table Featuring Compound Specific Details^a^

a Reaction conditions in the scheme: (i) 3.5 mol % NaHSO_4_·H_2_O, 0.2 mbar, 90–100 °C, 2–5 h; (ii) Jones’ reagent, THF/EtOAc/acetone, room temperature (RT), 1.5–4 h.

#### Structural Characterization

**Figure 2:**
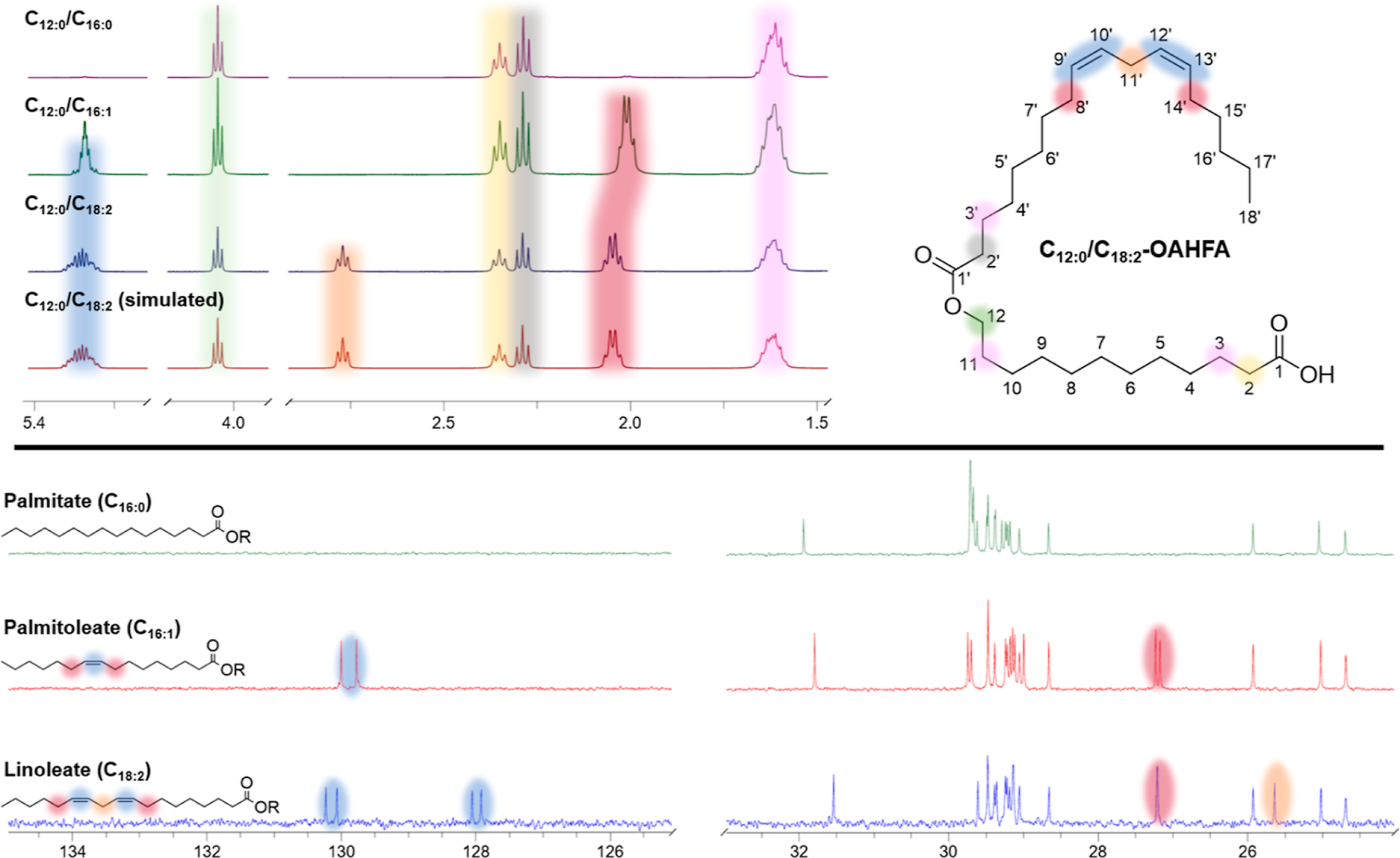
Comparison of selected ^1^H and ^13^C NMR chemical shifts of fully saturated (C_16:0_), monounsaturated (C_16:1_) and diunsaturated (C_18:2_) acyl groups (as well as some other key signals). Top left panel: ^1^H NMR chemical shifts of acyl groups with different degrees of unsaturation (from top downward: C_16:0_ → C_16:1_ → C_18:2_) are shown together with one further example highlighting the accuracy of the QMSA (measured vs simulated ^1^H NMR spectra of the C_12:0_/C_18:2-_OAHFA). Top right panel: the numbering of the C_12:0_/C_18:2_-OAHFA is shown with selected positions highlighted in colors matching their appearance in the spectra. Bottom panel: the ^13^C NMR spectra of the different acyl groups are shown (from top downward: C_16:0_ → C_16:1_ → C_18:2_) and key chemical shifts which can be used for rough grouping of acyl chains are color coded.

Structural characterization of the purified reaction products **1**–**16** was performed by high resolution mass spectrometry (HRMS) and nuclear magnetic resonance (NMR) spectroscopy. Detailed descriptions of our NMR characterization workflow, which is based upon a standard set of 1D- (^1^H, ^13^C) and 2D-NMR (DQF-COSY, Ed-HSQC, HMBC) experiments combined with quantum mechanical spectral analysis (QMSA) with the ChemAdder software,^28^ has been given in recent work.^17,25^ Through the use of the same protocols, the accurate chemical shifts and coupling constants (^1^H NMR spectra) could be reported for all well-separated signals in the ^1^H and ^13^C NMR spectra (see example in Figure 2). Moreover, the simulated accurate coupling constants were utilized to verify that the *Z*-configuration in the double bonds remained intact during the reactions. With access to detailed NMR-data on all common acyl groups in TFLL OAHFAs, including oleate groups from earlier work,^13^ we decided to compare their ^1^H and ^13^C NMR chemical shifts to identify potential signals which could enable rough assessment of their corresponding amounts in for example future NMR-based lipidomic profiling studies. While there are several characteristic ^1^H and ^13^C NMR signals for each of the acyl groups when analyzed in pure form, there are only a handful which allow grouping of the acyl chains into patterns that might be used for rough assessment of eventual mixtures, i.e., signals that are not directly overlapping in the ^1^H or ^13^C NMR spectra (see Figure 2).

In the ^1^H NMR spectra, characteristic chemical shifts for the linoleoyloxy group, which are not overlapping with signals from the other acyl groups, can be found at 2.77 ppm (dddd, H-11′) and 2.05 ppm (ddt, H-8′ and ddt, H-14′). The oleoyloxy and palmitoleoyloxy groups give rise to similar ^1^H NMR spectra and must be assessed as a joint group. The protons in close proximity to the alkene appear sufficiently upfield from the ones in the linoleoyloxy group (2.01 ppm; ddt, H-8′ and ddt, H-11′) thereby providing a potential opportunity for rough assessment of their combined amounts. The stearoyloxy and palmitoyloxy groups do not give rise to unique signals which could be utilized to provide a rough estimate of their amount per se. However, the ^1^H NMR signals which appear at an identical chemical shift in all the studied acyl groups could be utilized to provide a rough assessment of the amount of the stearoyloxy and palmitoyloxy groups since the amounts of the linoleoyloxy, oleoyloxy and palmitoyloxy can be deducted based on the integral values of the signals given above. The signals which appear at an identical chemical shift regardless of acyl group are the H-2′ which gives a t at 2.29 ppm and the terminal CH_3_ which appears as a t at 0.88 ppm.

In the ^13^C NMR spectra, the C-11′ at 25.8 ppm in the linoleoyloxy group was distinct from the signals observed in the other acyl groups. In addition, the linoleoyloxy group could be distinguished from the oleoyloxy and palmitoleoyloxy chains since the chemical shifts of the alkenes differ. In the linoleoyloxy group, the C-12′ and C-13′ appear at 128.1 and 130.2 ppm whereas the C-9′ and C-10′ appear at 130.4 and 128.2 ppm. These signals do not overlap with the C-9′ and C-10′ of the palmitoleoyloxy and oleoyloxy groups which appear at 130.1 and 129.9 ppm. In a similar fashion as above, the stearoyloxy and palmitoyloxy groups did not give rise to unique signals in the ^13^C NMR spectra. However, the signals which occur at identical chemical shifts in all the acyl groups could be utilized to assess the relative amount of these species as the contributions of the linoleoyloxy, palmitoleoyloxy and oleoyloxy would be known. The signals which occur at identical chemical shifts in the ^13^C NMR spectra are the C-2′ at 34.6 ppm, the C-3′ at 25.2 ppm and the terminal CH_3_ at 14.3 ppm.

In conclusion, the protocols we have developed for NMR spectroscopic characterization of TFLL lipids are sound and the ^1^H and ^13^C NMR spectra could be characterized at a high level. The generated ^1^H and ^13^C NMR data of distinct acyl groups could in principle be used as a base for future lipidomic profiling studies, but the practicality of the approach will need to be carefully assessed separately to verify its suitability for early stage profiling of meibum/tear fluid composition. It is possible that NMR will not be able to match the sensitivity and accuracy of the widely employed mass spectrometric protocols.^6,8,10,11^ Nevertheless, the intention was to map the biophysical properties of the representative OAHFA-analogues, i.e., compounds **9**–**16**, and thus we continued with work aimed toward this goal.

### Biophysical Profiling

To be able to draw meaningful conclusions on the roles and contributions of OAHFAs to a functioning TFLL, the structure–property profiles of individual species must first be thoroughly mapped. While we in this work assess the properties of these species from a TFLL point-of-view, the behavior of the lipid films (i.e., Langmuir monolayer studies) could also have been discussed from a broader surface science perspective.^29,30^ Within the TFLL context, the OAHFA species are referred to as polar lipids and they are net contributors to the structure and function of the polar lipid layer which is in direct contact with the aqueous tear film layer. We have in the past reported on the properties of a wide range of oleoyloxy containing OAHFAs and these studies will here be used as a valuable reference point for the assessment of other acyl groups.^13^ Most importantly, identical experimental protocols were employed, allowing direct comparison of the effects of distinct acyl groups, degree of saturation and chain length. The biophysical profiling of the OAHFA library was divided into two parts: measurement of melting points and studies on the properties of Langmuir monolayers.

#### Melting Points

The melting points of tear film lipids form a basic parameter which can be utilized to predict the behavior of their films at the aqueous interface. In previous reports, researchers have suggested that understanding the link between melting points and film properties is the key to unlocking the secrets of the TFLL.^31,32^ In our recent studies on the effects of branching in TFLL wax and cholesteryl esters (WEs and CEs), we have proven that this approach is indeed valuable.^25,33^ On a general level, the melting points are considered important for two reasons: (1) they affect the fluidity of meibum which is important both during secretion from the meibomian glands and during spreading of the film at the ocular surface, and (2) they affect the packing, structure and adaptive features of the TFLL film which is central to its role in maintenance of ocular surface health. In the current study, we measured the melting points of both OAHFAs **9**–**16** and their *O*-acyl-ω-hydroxy fatty alcohol (OAHFAl) counterparts **1**–**8**. This set of compounds enabled us to address the effect of terminal functional group, chain length and degree of saturation on the melting points. An overview of the determined melting points is provided in Table 2.

**Table 2:** Overview of the Determined Melting Points for OAHFAls and OAHFAs

parent chain	acyl group	OAHFAl (°C)	OAHFA (°C)
C_12:0_	C_16:0_	63.3	66.7
C_12:0_	C_16:1_	33.4	36.8
C_12:0_	C_18:0_	66.6	71.2
C_12:0_	C_18:2_	26.0	30.3
C_20:0_	C_16:0_	72.4	76.8
C_20:0_	C_16:1_	57.0	61.0
C_20:0_	C_18:0_	77.9	81.3
C_20:0_^a^	C_18:1_	59	62
C_20:0_	C_18:2_	51.0	53.6
C_20:1_^b^	C_18:1_		30.1
C_29:1_^c^	C_18:1_	49.2	53.5

a Value reported by Bland et al.^13^

b Value reported by Viitaja et al.^17^

c Value reported by Viitaja et al.^15^

The following trends could be uncovered:

The difference in the melting points between the OAHFAls and their corresponding OAHFAs was found to be marginal. In all substrates studied herein, as well as in our earlier work,^15,17^ the OAHFAs have a melting point which is ∼3–5 °C higher than that of the OAHFAls. The effect of the terminal functional group (alcohol vs carboxylic acid) in these species seems minimal compared to the effects of chain length and degree of saturation.

The effects of chain length and degree of saturation on the melting points of OAHFAls and OAHFAs were found to be heavily dependent on each other. Therefore, they were assessed together. In more detail, in the species based on the shorter C_12:0_ parent chain, the effects of introducing 1–2 *Z*-alkene functionalities resulted in significant decreases in the melting points. The differences between the species bearing the C_16:0_ or C_16:1_ acyl chains were ∼30 °C whereas the difference between the species bearing the C_18:0_ or C_18:2_ acyl chains were ∼40 °C. When the species with the longer C_20:0_ parent chain were analyzed, the influence of the *Z*-alkene functionalities remained considerable although the effect was less pronounced. In this series, the introduction of a *Z*-alkene in the C_16:0_ chain was accompanied by a 15 °C decrease in the melting point whereas the introduction of two *Z*-alkenes in the C_18:0_ chain was accompanied by a 27 °C decrease in melting point.

In both series (longer vs shorter parent chain lengths), the effect of increasing the chain length of the acyl group by 2 carbon atoms (C_16:0_ vs C_18:0_) was accompanied by a 4–5 °C increase in the melting point. The effects of increasing the parent chain length by 8 carbon atoms was accompanied by a 10 °C increase in the melting point for saturated species and a 24 °C increase in the melting point for unsaturated species.

Altogether, several distinct trends could be observed regarding the effects of chain length, degree of saturation and terminal functional group on the melting points of both OAHFAls and OAHFAs. The trends uncovered fit well with the observed melting point differences witnessed in our previous work on C_20:1_/C_18:1_ (mp ∼ 30.1 °C) and C_29:1_/C_18:1_-OAHFA (mp ∼ 53.5 °C). It should be noted that the location of the double bonds in the species do affect the melting points and the previously synthesized species display similar melting points to the C_12:0_/C_18:2_ (in the case of C_20:1_/C_18:1_) and C_20:0_/C_18:2_ (in the case of C_29:1_/C_18:1_) OAHFAs in the current set. Nevertheless, the melting point range of the representative OAHFA library (30.3–81.3 °C) covers an essential part of the melting point spectrum displayed by the naturally occurring OAHFAs (C_24:1_/C_18:1_–C_34:1_/C_18:1_). Thus, a solid foundation for continued studies focusing on the extent to which the melting points of individual OAHFAs correlate with/predict their surface behavior was established.

#### Langmuir Monolayer Studies

In the Langmuir monolayer studies we decided to focus on the properties displayed by the OAHFA species **9**–**16**. Since these species are expected to be an integral part of the polar lipid layer and in direct contact with the aqueous tear film, we decided to emulate this situation using our standard Langmuir–Blodgett trough setup. Shortly, the OAHFA species dissolved in chloroform (5 mM solutions) were administered onto the aqueous subphase (PBS-buffer; 140 mM NaCl, 3 mM KCl, 10 mM phosphate buffer, pH 7.4) and the biophysical profiling was performed at ocular surface temperature (35 °C). The biophysical profiling studies included: measurement of the surface pressure and surface potential isotherms over compression–expansion cycles, imaging of the film structure by Brewster angle microscopy (BAM), measurement of the evaporation resistance at selected surface pressures, and preliminary profiling of the viscoelastic properties of the films formed. Altogether, these experiments were thought to give a strong base for assessing how the acyl groups might affect distinct properties in the OAHFA species. We will first go through the results in the C_12:0_ and C_20:0_ series separately and then provide a short summary placing the findings into a wider perspective.

#### Observation in the C_12:0_ Series

**Figure 3:**
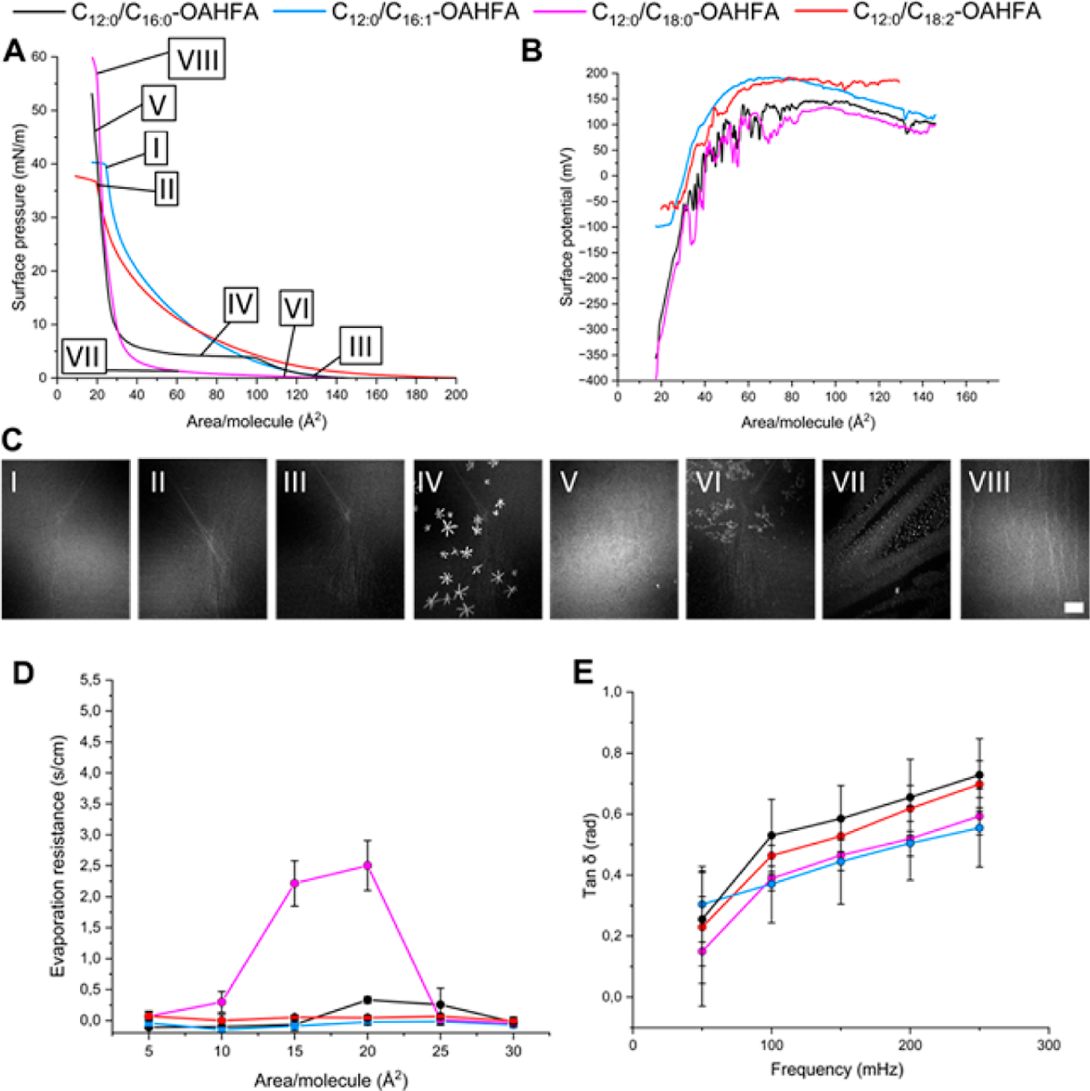
Excerpt of biophysical profiling data for the shorter C_12:0_-species **9**–**12** (**9**: C_12:0_/C_16:0_, **10**: C_12:0_/C_16:1_, **11**: C_12:0_/C_18:0_, **12**: C_12:0_/C_18:2_). (A) Surface pressure isotherms are given as a function of the mean molecular area. The surface pressure isotherms are numbered (I–VIII) and the numbers are utilized to create a link to the BAM-images displayed. (B) The surface potential isotherms are given as a function of the mean molecular area. (C) The BAM-images (I–VIII) highlight the visual appearance of the lipid films at selected mean molecular areas. The scale bar depicts 500 μm in the BAM images. (D) The evaporation resistance is given as a function of mean molecular area on the s/cm scale. (E) Changes in the loss tangent are displayed as a function of frequency.

We began our assessment by studying the properties of the shorter OAHFA species **9**–**12**. An overview of the results from the biophysical profiling studies can be found in Figure 3. Based on the melting point studies performed, we expected to see a difference in the surface behavior of the saturated and unsaturated species. The films of the unsaturated species bearing a C_16:1_ or C_18:2_ acyl group remained in the liquid phase throughout the compression–expansion cycle (Figure 3, BAM-images I and II) and the film was found to collapse at pressures close to the physiological ones (30–45 mN/m, see Figure 3A, surface pressure isotherm). The films of the saturated species bearing a C_16:0_ or C_18:0_ acyl group went from liquid to solid at low surface pressures (see BAM-images III–V for **9**, and VI–VIII for **11** in Figure 3C) and the films were found to be stable at surface pressures >50 mN/m. Altogether, the surface pressure isotherms were similar to those observed in our earlier work and the conformations adopted by the lipid species as a result of increased pressure and tighter packing can be thought to follow similar trends. In other words, the orientation of the lipids change gradually from lying flat at the aqueous surface to a more upright conformation. Further evidence of this gradual change in orientation is seen from the surface potential isotherms. While the surface potential isotherms were found to follow similar trends for all these species (Figure 3B), the species containing unsaturated acyl groups did display a slightly higher effective molecular dipole moment prior to the conformational change. Differences were also noted at the end point of the surface potential measurement. The saturated species displayed a more negative surface potential than the unsaturated ones. This difference is likely due to a tighter and more ordered packing of the saturated species, which are in a solid phase.

Interestingly, the unsaturated species were found to behave in a similar fashion as the previously studied C_20:1_/C_18:1_-OAHFA analogue.^17^ Coincidence or not, the melting points of these species are all in the range of 30–37 °C. The C_20:1_/C_18:1_-OAHFA represents the lower end of the chain length spectrum in naturally occurring TFLL OAHFAs. Thus, the unsaturated species featuring the C_12:0_ parent chain can be considered as good model compounds at the lower end of the OAHFA spectrum. Studies on TFLL composition and function have indicated that there is a shift toward shorter OAHFA species as a result of ocular surface diseases such as dry eye disease (DED).^34^ Since more than 80% of DED-cases are accompanied by an increased evaporation rate of aqueous tear fluid,^35^ we decided to elucidate whether distinct acyl groups in the shorter species could impact the evaporation resistance of the film formed. We would like to note that through our earlier work on individual OAHFA species as well as different types of lipid assemblies,^13,17,33^ a sound framework for assessing when evaporation resistance can be expected and a reference scale was available. A solid monolayer structure is required to reach a decent evaporation resistance in a Langmuir monolayer. From the shorter OAHFA series studied, only OAHFAs **9** and **11** showed the required film structure in the BAM-images, although at ocular surface temperature (35 °C) and pressure (30–45 mN/m), none of the shorter OAHFAs **9**–**12** displayed promising evaporation resistant properties (Figure 3D). Upon further compression to mean molecular areas of ∼20 Å^2^/molecule, the films of the unsaturated species collapsed whereas a decent evaporation resistance of 2.5 s/cm could be obtained for **11**. Thus, differences in the behavior of the distinct acyl groups were apparent.

Another important factor relating to the function of the TFLL, is its adaptive features over compression–expansion cycles. The Langmuir setup is not ideal for addressing these factors at a detailed level, but it is sufficient for a preliminary assessment of the viscoelastic properties of the lipid films. These studies were performed using an oscillating barrier protocol over the frequency range of 100–250 mHz. Information on the loss modulus *G*″ and storage modulus *G*′, i.e., the viscous vs elastic components of the complex modulus G*, could be obtained and further utilized to provide a rough estimate of the nature of the films and their adaptive features. Plotting the loss tangent as a function of frequency (Figure 3E) indicates that all C_12:0_-species display viscoelastic behavior and a gradual shift toward increased viscosity is noted upon an increase in the frequency. With respect to previous studies on TFLL model systems,^36^ OAHFAs with similar properties as the C_12:0_-species would be net contributors to film elasticity.

#### Observations in the C_20:0_ Series

**Figure 4:**
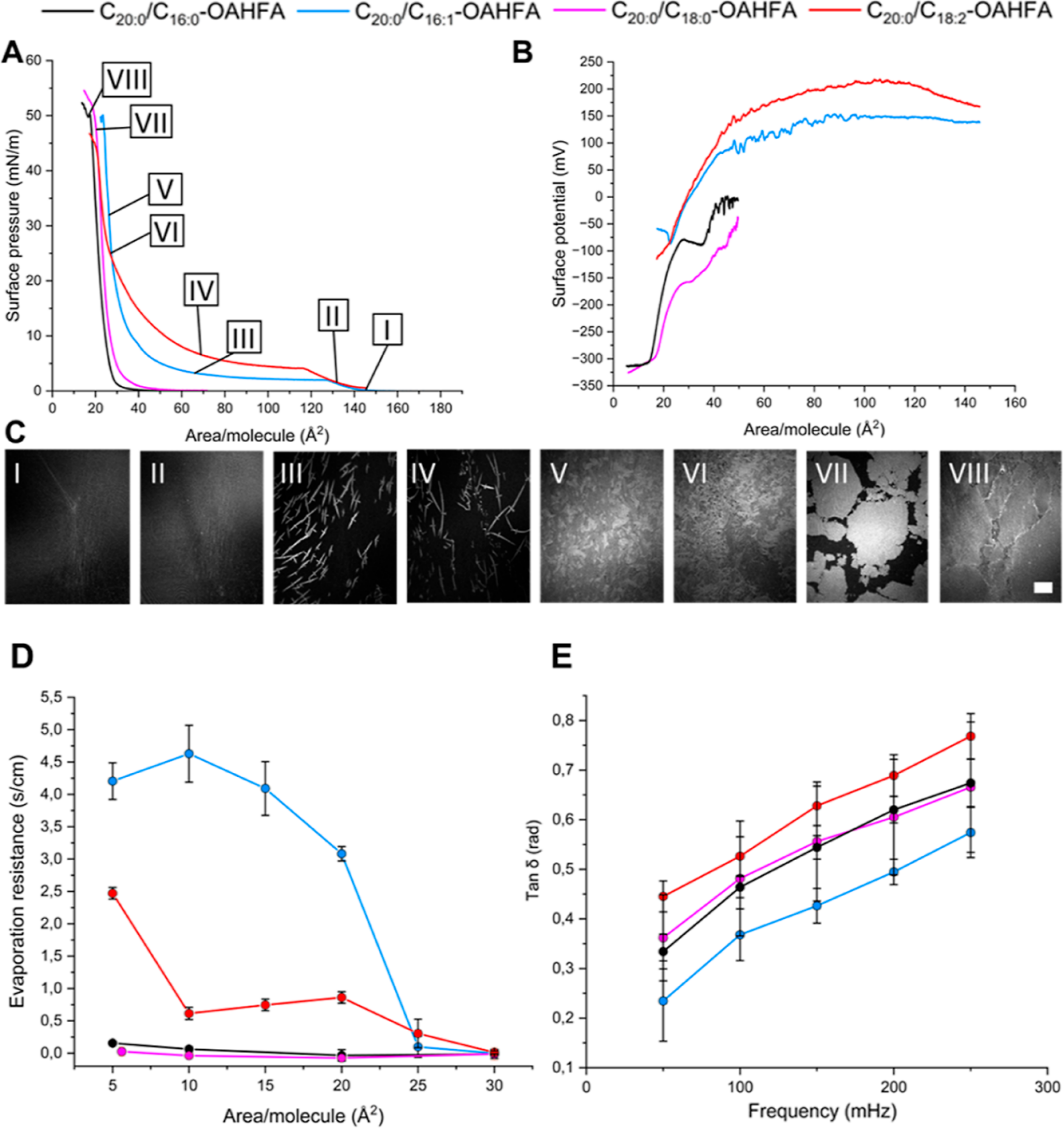
Excerpt of biophysical profiling data for the shorter C_20:0_-species **13**–**16** (**13**: C_20:0_/C_16:0_, **14**: C_20:0_/C_16:1_, **15**: C_20:0_/C_18:0_, **16**: C_20:0_/C_18:2_). (A) Surface pressure isotherms are given as a function of the mean molecular area. The surface pressure isotherm is numbered (I–VIII) and the number are utilized to create a link to the BAM-images displayed. (B) The surface potential isotherms are given as a function of the mean molecular area. (C) The BAM-images (I–VIII) highlight the visual appearance of the lipid films at selected mean molecular areas. The scale bar depicts 500 μm in the BAM images. (D) The evaporation resistance is given as a function of mean molecular area on the s/cm scale. (E) Changes in the loss tangent are displayed as a function of frequency.

The melting points of the longer OAHFA species **13**–**16** were all in the range of 54–81 °C. These melting points cover the range expected for the longer (and most abundant) naturally occurring OAHFAs but also shorter species bearing saturated acyl groups as exemplified by structures **9** and **11** analyzed above. The Langmuir monolayer studies were therefore expected to complement well the ones performed on the shorter series based on the C_12:0_ parent chain. An overview of the results from the biophysical profiling of the C_20:0_ series can be found in Figure 4.

On a general level, the surface pressure and potential isotherms followed similar trends as conveyed above for the OAHFAs **9**–**12**. Although, deviations were observed. For example, the surface pressure lift-off area was found to be similar for the species bearing an unsaturated acyl group whereas the surface pressure lift-off for the species bearing a saturated acyl group occurred at a significantly lower area/molecule (Figure 4A). The same trends could partly be observed in the shorter species studied, but the effect was more pronounced in the longer series. The phase transitions occurring at low surface pressures for species **14** and **16** was accompanied by the formation of crystals (see BAM-images I–III for **14**, and IV–VI for **16** in Figure 4C) which gradually grew into a solid monolayer upon an increase in surface pressure. Overall, the surface behavior of these species were similar to that of the previously reported C_20:0_/C_18:1_-OAHFA.^13^ Interestingly, the C_20:0_/C_18:2_-OAHFA (**16**) did form a solid monolayer whereas the same chain length species C_20:1_/C_18:1_-OAHFA, bearing a different placement of the double bonds, remained in the liquid state throughout the measurements in our previous work.^17^ Thus, the degree of saturation is an unreliable indicator which cannot directly be utilized to predict surface behavior. In this case, the melting point differences of 53.6 vs 30.3 °C could be considered a more accurate measure. The surface behavior of the OAHFA species **13** and **15**, bearing a saturated acyl group, differed from species **14** and **16** as they formed a solid condensed monolayer straight away as indicated by the high intensity areas observed in the BAM images (VII and VIII, Figure 4C).

In the surface potential isotherms, similar trends as those discussed above were observed. Therefore, these species are expected to undergo similar changes in orientation as noted in previous work.^13^ For the unsaturated species, there is an addition to the molecular dipole moment based on the alkene moiety present in the acyl group. Apart from this, the surface potential data could be utilized to analyze whether there is a change in the orientation of the molecules prior to phase transitions occurring or upon formation of a solid phase. As an example, the surface pressure lift-off is not accompanied by a significant change in the effective dipole moment, indicating that the dipole moments are not reconfigured at this stage whereas a significant change can be observed at higher surface pressures due to the formation of a solid monolayer (Figure 4C). The end points of the surface potential isotherms differ in the distinct species which indicates that the acyl groups inflict a change in the tilt angles of the OAHFAs at the interface (assuming that the interactions of the carboxylic acid residue at the interface is similar in all cases), but more insights on these features will require additional gracing incidence X-ray diffraction studies in the future.^37^

In addition to the factors discussed above, the overall chain lengths were found to affect the stability of the lipid films. This was apparent since the films formed by the longer OAHFAs **13**–**16** were all found to be stable at ocular surface pressures of 30–45 mN/m. The stability of these films, and the solid monolayers formed, indicated that these species could have decent evaporation resistant properties. However, at ocular surface temperature (35 °C) and pressure (30–45 mN/m), only the film formed by C_20:0_/C_16:1_-OAHFA (**14**) displayed a decent evaporation resistance of ∼3.3 s/cm (Figure 4D). This value is similar to the one earlier reported for the C_20:0_/C_18:1_-OAHFA analogue.^13^ Based on the screening performed, the majority of OAHFA species are not expected to display significant evaporation resistant properties on their own at ocular surface temperature and pressure. However, in specific OAHFA species which have an optimal chain length and degree of saturation such features can arise. In these cases, the overall properties of the molecules need to be carefully balanced as they are dependent on the structural contributions of both the parent chain and the acyl group. Based on the results obtained, we would expect films formed by the most abundant TFLL OAHFAs, i.e., the C_32:1_/C_18:1_- and the C_34:1_/C_18:1_-OAHFAs, to display antievaporative properties.

Our assessment of the viscoelastic properties of the C_20:0_ series revealed that these molecules behave in a similar fashion as the shorter C_12:0_ series. In other words, an increase in frequency is accompanied by a clear shift toward more viscous behavior (Figure 4E). Interestingly, in the C_20:0_ series, the most viscous behavior was observed for the C_20:0_/C_18:2_-OAHFA. These findings deviate from the patterns observed in the C_12:0_ series. Therefore, while the OAHFA analogues studied herein would suggest that these species are net contributors to film elasticity, a trend could not be uncovered which would allow more detailed predictions of the rheology of OAHFA monolayers based on the chemical structure displayed.

#### Effects of Acyl Groups on the Properties of OAHFAs, Take-Away Message

Rather than just considering the effects of the acyl group on distinct OAHFA species, this study indicates that the effects of acyl groups cannot be analyzed in vacuum but needs to be assessed together with the overall chain length and molecular structure of the lipid species in question. Nevertheless, some factors are clearly affected by the acyl groups. For example, saturated vs unsaturated acyl groups will affect the molecular dipole moments and the tilt-angle at the aqueous interface simultaneously affecting the properties of the polar lipid layer. In addition, the acyl group will affect the viscoelastic properties of the lipids and thus the adaptability of the polar lipid layer. It should be noted that these effects become more pronounced in species with longer chain lengths than in species with shorter chain lengths. The distinct acyl groups also affect the melting points of the lipid species to a degree which can now be predicted quite accurately based on our profiling studies. While the melting points were once suggested to be the key for predicting the surface behavior of the OAHFA species (as well as other TFLL lipids), our results show that this is not a straightforward concept that can explain all features displayed by the tear film OAHFAs. However, the melting points can be utilized for rough division of the OAHFA species into three categories.

OAHFA species with melting points in the range of 30–37 °C (or below) can be expected to remain in a liquid phase and the films formed by these species in pure form are likely to collapse at ocular surface pressures or below.

OAHFA species with melting points in the range of 54–67 °C (the category to which the most abundant TFLL OAHFAs are likely to belong) form stable films at ocular surface temperature and pressure. Moreover, these films are likely to undergo liquid to solid phase transitions. Films of specific OAHFA species in this melting point range may display evaporation resistant features although not all factors affecting such capabilities are yet identified. Nevertheless, such properties seem to arise when there is an optimal balance between spreadability and the capability to form a densely packed solid monolayer. In the OAHFA species screened to date, only films formed by the C_20:0_/C_18:1_, C_20:0_/C_16:1_ and C_29:1_/C_18:1_ OAHFAs have displayed modest evaporation resistance at ocular surface temperature and pressure. Moreover, our previous studies, as well as those by other teams, have indicated that the source of TFLL evaporation resistance is more likely to stem from the collaborative action of distinct lipid classes or interactions taking place between the lipid layer and other biomolecules.^38^ Even in such a scenario, the most abundant TFLL OAHFAs are likely to play an active role.

OAHFAs with melting points in the range of 71–81 °C (or above) can be expected to form solid monolayers directly in a distinct fashion than those observed for the category above. While these species form a solid monolayer, which is a prerequisite for evaporation resistance, none of the species studied in this range have displayed evaporation resistant properties at ocular surface temperature and pressure.

Based on the rheological measurements reported here and data from our previous study,^15^ the lipid films formed by long-chain OAHFA species are more elastic than their shorter chain analogues. This may improve the adaptability of the lipid films and be a reason behind the evolutionary drive toward the ultralong OAHFA species found in the TFLL today.

## Experimental Section

### General Information on the Synthesis and Characterization of Lipid Species

All chemicals were purchased from commercial sources (Acros Organics, Geel, Belgium; Honeywell, Seelze, Germany; Nu-Chek Prep, Elysian MN, USA; Fluorochem, Hadfield, UK; Merck, Darmstadt, Germany) and used as such unless otherwise stated. TLC was performed on aluminum sheets precoated with silica gel 60 F254 (Merck) and spots were visualized by spraying the plates with a 5:1 MeOH/H_2_SO_4_-solution followed by charring. Flash chromatography was carried out on silica gel 40 (40–63 μm, Merck). NMR spectra were recorded on a Bruker Avance III instrument operating at 500 MHz (^1^H = 499.82 MHz, ^13^C = 125.68 MHz). The probe temperature was kept at 25 °C unless otherwise stated. The NMR spectra were processed using the Bruker TopSpin 4.3.0 software, and the QMSA was performed with the ChemAdder (Spin Discoveries Inc., Kuopio, Finland) software.^28^ Chemical shifts are expressed on the ppm scale using tetramethylsilane (TMS; δ_H_ 0 ppm; δ_C_ 0 ppm) or residual solvent signals of CHCl_3_ (δ_H_ 7.26 ppm; δ_C_ 77.16 ppm) as the internal reference. Coupling constants are given in Hz and provided only once when first encountered. The coupling patterns are given as singlet (s), doublet (d), triplet (t), multiplet (m), etc. A standard set of 1D (^1^H, ^13^C, and TOCSY) and 2D (DQF-COSY, Ed-HSQC, HMBC) NMR experiments were recorded with pulse sequences provided by the instrument manufacturer. High resolution mass spectra were recorded on a Bruker micro Q-TOF mass spectrometer (Bruker Daltonics, Bremen, Germany) with ESI (electrospray ionization) operated in positive mode. Melting points were determined on a Mettler Toledo MP50 (Columbus, OH, USA) melting point device with an accuracy of 0.1 °C. Three measurements were performed and the relative standard deviation was found to be within a 1% margin. Please note that both in the substrate specific analytical details below and in Table 2, only one value is given.

#### General Procedure for Synthesis of OAHFAls

The corresponding fatty acid (0.9–1.1 equiv) and NaHSO_4_·H_2_O (3.5 mol %) were added to a flask containing the corresponding diol (1.0 equiv). The reaction mixture was heated at 90–100 °C (the reaction mixture was in the liquid state) with stirring under vacuum for 2–5 h. The reaction mixture was cooled to RT, and the crude product was purified by column chromatography (*n*-hexane/EtOAc 9:1 → 4:1), and dried in vacuo to obtain the pure OAHFAl.

#### General Procedure for Synthesis to OAHFAs

To the corresponding OAHFAl (1 equiv) dissolved in a 4:2:1–1:1:1 mixture (depending on the substrate) of THF/acetone/EtOAc was added Jones’ reagent (CrO_3_ 2.0 mol/L in aq H_2_SO_4_, 2.2–3.6 equiv) at RT and the reaction progress was monitored by TLC. Upon disappearance of the starting material spot, the reaction was quenched through the addition of isopropanol (4 mL/100 mg OAHFAl). The resulting mixture was filtered through Celite and the Celite was properly washed with Et_2_O (300 mL/100 mg OAHFAl). When required, the filtrate was further washed with brine (2 × 40 mL/100 mg OAHFAl), the organic phase was separated, dried over anhydrous Na_2_SO_4_, filtered and concentrated. The crude product was purified by column chromatography (*n*-hexane/EtOAc/AcOH 9:1:0.01 → 7:3:0.01) and dried in vacuo to obtain the corresponding OAHFA.

### Substrate Specific Analytical Data

#### 12-(Palmitoyloxy)dodecan-1-ol (**1**)

Compound **1** was synthesized according to the general procedure for synthesis of OAHFAls. The following amounts of the reagents were used: palmitic acid (443.5 mg, 1.73 mmol, 1.0 equiv), NaHSO_4_·H_2_O (8.4 mg, 0.06 mmol, 3.5 mol %), and 1,12-dodecanediol (350.0 mg, 1.73 mmol, 1.0 equiv). The reaction temperature was 100 °C and the reaction time 2 h. A white solid was received after purification (349.1 mg, 46%). *R*_*f*_ = 0.17 (*n*-hexane/EtOAc 4:1), mp 63.3 °C.

^1^H NMR (499.82 MHz, 25 °C, CDCl_3_): δ 4.05 (t, 2H, *J*_12,11_ = 6.6 Hz, H-12), 3.64 (dt, 2H, *J*_1,1-OH_ = 5.3, *J*_1,2_ = 6.6 Hz, H-1), 2.29 (t, 2H, *J*_2′,3′_ = 7.5 Hz, H-2′), 1.62 (tt, 2H, *J*_11,10_ = 6.9 Hz, H-11), 1.60 (tt, 2H, *J*_3′,4′_ = 7.3 Hz, H-3′), 1.57 (tt, 2H, *J*_2,3_ = 7.5 Hz, H-2), 1.38–1.20 (m, 41H, 1-OH, H-3–H-10, H-4′–H-15′) and 0.88 (t, 3H, *J*_18′,17′_ = 7.5 Hz, H-16′) ppm.

^13^C NMR (125.68 MHz, 25 °C, CDCl_3_): δ 174.2 (C-1′), 64.5 (C-12), 63.2 (C-1), 34.6 (C-2′), 33.0 (C-2), 32.1 (C-14′), 29.8–29.3 (C-4–C-9, C-4′–C-13′), 28.8 (C-11), 26.1 (C-10), 26.0 (C-3), 25.2 (C-3′), 22.8 (C-15′) and 14.3 (C-16′) ppm.

HRMS (EI): *m*/*z* calcd for C_28_H_56_O_3_Na [M + Na]^+^, 463.4122; found, 463.4109.

#### 12-(Palmitoleoyloxy)dodecan-1-ol (**2**)

Compound **2** was synthesized according to the general procedure for synthesis of OAHFAls. The following amounts of the reagents were used: palmitoleic acid (440.1 mg, 1.73 mmol, 1.0 equiv), NaHSO_4_·H_2_O (8.4 mg, 0.06 mmol, 3.5 mol %), and 1,12-dodecanediol (350.0 mg, 1.73 mmol, 1.0 equiv). The reaction temperature was 99 °C and the reaction time 2 h. A white solid was received after purification (0.2270 g, 30%). *R*_*f*_ = 0.26 (*n*-hexane/EtOAc 4:1), mp 33.4 °C.

^1^H NMR (499.82 MHz, 25 °C, CDCl_3_): δ 5.35 (dtt, 1H, *J*_9′,11′_ = −1.7, *J*_9′,8′_ = 7.3, *J*_9′,10′_ = 10.5 Hz, H-9′), 5.34 (dtt, 1H, *J*_10′,8′_ = −1.7, *J*_10′,11′_ = 7.2 Hz, H-10′), 4.05 (t, 2H, *J*_12,11_ = 6.8 Hz, H-12), 3.64 (t, 2H, *J*_1,2_ = 6.7 Hz, H-1), 2.29 (t, 2H, *J*_2′,3′_ = 7.6 Hz, H-2′), 2.01 (ddt, 2H, *J*_8′,7′_ = 7.3 Hz, H-8′), 2.01 (ddt, 2H, *J*_11′,12′_ = 6.7 Hz, H-11′), 1.62 (tt, 2H, *J*_11,10_ = 7.1 Hz, H-11), 1.61 (tt, 2H, *J*_3′,4′_ = 6.7 Hz, H-3′), 1.56 (tt, 2H, *J*_2,3_ = 6.8 Hz, H-2), 1.38–1.20 (m, 32H, H-3–H-10, H-4′–H-7′, H-12′–H-15′) and 0.88 (t, 3H, *J*_16′,15′_ = 6.9 Hz, H-16′) ppm.

^13^C NMR (125.68 MHz, 25 °C, CDCl_3_): δ 174.1 (C-1′), 130.1 (C-9′), 129.9 (C-10′), 64.5 (C-12), 63.2 (C-1), 34.5 (C-2′), 33.0 (C-2), 31.9 (C-14′), 29.9–28.8 (C-4–C-9, C-4′–C-7′, C-12′–C-13′), 28.8 (C-11), 27.4–27.3 (C-8′, C-11′), 26.1 (C-10), 25.9 (C-3), 25.2 (C-3′), 22.8 (C-15′) and 14.2 (C-16′) ppm.

HRMS (EI): *m*/*z* calcd for C_28_H_54_O_3_Na [M + Na]^+^, 461.3965; found, 461.4001.

#### 12-(Stearoyloxy)dodecan-1-ol (**3**)

Compound **3** was synthesized according to the general procedure for synthesis of OAHFAls. The following amounts of the reagents were used: stearic acid (541.3 mg, 1.90 mmol, 1.1 equiv), NaHSO_4_·H_2_O (8.4 mg, 0.06 mmol, 3.5 mol %), and 1,12-dodecanediol (350.0 mg, 1.73 mmol, 1.0 equiv). The reaction temperature was 90 °C and the reaction time 3 h. A white solid was received after purification (409.4 mg, 50%). *R*_*f*_ = 0.26 (*n*-hexane/EtOAc 4:1), mp 66.6 °C.

^1^H NMR (499.82 MHz, 25 °C, CDCl_3_): δ 4.05 (t, 2H, *J*_12,11_ = 6.7 Hz, H-12), 3.64 (dt, 2H, *J*_1,1-OH_ = 5.3, *J*_1,2_ = 6.8 Hz, H-1), 2.29 (t, 2H, *J*_2′,3′_ = 7.5 Hz, H-2′), 1.62 (tt, 2H, *J*_11,10_ = 6.8 Hz, H-11), 1.60 (tt, 2H, *J*_3′,4′_ = 7.3 Hz, H-3′), 1.56 (tt, 2H, *J*_2,3_ = 6.9 Hz, H-2), 1.38–1.22 (m, 44H, H-3–H-10, H-4′–H-17′), 1.21 (t, 1H, 1-OH) and 0.88 (t, 3H, *J*_18′,17′_ = 6.8 Hz, H-18′) ppm.

^13^C NMR (125.68 MHz, 25 °C, CDCl_3_): δ 174.2 (C-1′), 64.5 (C-12), 63.2 (C-1), 34.6 (C-2′), 33.0 (C-2), 32.1 (C-16′), 29.8–29.3 (C-4–C-9, C-4′–C-15′), 28.8 (C-11), 26.1 (C-10), 26.0 (C-3), 25.2 (C-3′), 22.8 (C-17′) and 14.3 (C-18′) ppm.

HRMS (EI): *m*/*z* calcd for C_30_H_60_O_3_Na [M + Na]^+^, 491.4406; found, 491.4406.

#### 12-(Linoleoyloxy)dodecan-1-ol (**4**)

Compound **4** was synthesized according to the general procedure for synthesis of OAHFAls. The following amounts of the reagents were used: linoleic acid (533.6 mg, 1.90 mmol, 1.1 equiv), NaHSO_4_·H_2_O (8.4 mg, 0.06 mmol, 3.5 mol %), and 1,12-dodecanediol (350.0 mg, 1.73 mmol, 1.0 equiv). The reaction temperature was 90 °C and the reaction time 3 h. A white solid was received after purification (345.1 mg, 43%). *R*_*f*_ = 0.43 (*n*-hexane/EtOAc 4:1), mp 26.0 °C.

^1^H NMR (499.82 MHz, 25 °C, CDCl_3_): δ 5.38 (dtt, 1H, *J*_9′,11′_ = −1.7, *J*_9′,8′_ = 7.2, *J*_9′,10′_ = 10.6 Hz, H-9′), 5.37 (dtt, 1H, *J*_13′,11′_ = −1.5, *J*_13′,14′_ = 6.9, *J*_13′,12′_ = 10.8 Hz, H-13′), 5.33 (dtt, 1H, *J*_10′,8′_ = −1.7, *J*_10′,11′_ = 6.7 Hz, H-10′), 5.33 (dtt, 1H, *J*_12′,14′_ = −2.0, *J*_12′,11′_ = 7.7 Hz, H-12′), 4.05 (t, 2H, *J*_12,11_ = 6.8 Hz, H-12), 3.64 (dt, 2H, *J*_1,1-OH_ = 4.7, *J*_1,2_ = 6.6 Hz, H-1), 2.77 (dddd, 2H, H-11′), 2.28 (t, 2H, *J*_2′,3′_ = 7.3 Hz, H-2′), 2.05 (ddt, 2H, *J*_8′,7′_ = 7.3 Hz, H-8′), 2.05 (ddt, 2H, *J*_14′,15′_ = 6.8 Hz, H-14′), 1.62 (tt, 2H, *J*_11,10_ = 7.1 Hz, H-11), 1.61 (tt, 2H, *J*_3′,4′_ = 7.3 Hz, H-3′), 1.56 (tt, 2H, *J*_2,3_ = 7.5 Hz, H-2), 1.39–1.22 (m, 31H, 1-OH, H-3–H-10, H-4′–H-7′, H-15′–H-17′) and 0.88 (t, 3H, *J*_18′,17′_ = 6.8 Hz, H-18′) ppm.

^13^C NMR (125.68 MHz, 25 °C, CDCl_3_): δ 174.1 (C-1′), 130.3 (C-9′), 130.2 (C-13′), 128.2 (C-10′), 128.0 (C-12′), 64.5 (C-12), 63.2 (C-1), 34.5 (C-2′), 32.9 (C-2), 31.7 (C-16′), 29.7–29.3 (C-4–C-9, C-4′–C7′, C-15′), 28.8 (C-11), 27.3 (C-8′, C-14′), 26.1 (C-10), 25.9 (C-3), 25.8 (C-11′), 25.1 (C-3′), 22.7 (C-17′) and 14.2 (C-18′) ppm.

HRMS (EI): *m*/*z* calcd for C_30_H_56_O_3_Na [M + Na]^+^, 487.4122; found, 487.4111.

#### 20-(Palmitoyloxy)eicosan-1-ol (**5**)

Compound **5** was synthesized according to the general procedure for synthesis of OAHFAls. The following amounts of the reagents were used: palmitic acid (1.02 g, 3.98 mmol, 1.0 equiv), NaHSO_4_·H_2_O (19.4 mg, 0.14 mmol, 3.5 mol %), and 1,20-eicosanediol^25^ (1.25 g, 3.97 mmol, 1.0 equiv). The reaction temperature was 95 °C and the reaction time 4 h. A white solid was received after purification (872.3 mg, 40%). *R*_*f*_ = 0.43 (*n*-hexane/EtOAc 4:1), mp 72.4 °C.

^1^H NMR (499.82 MHz, 25 °C, CDCl_3_): δ 4.05 (t, 2H, *J*_20,19_ = 6.7 Hz, H-20), 3.64 (t, 2H, *J*_1,2_ = 6.6 Hz, H-1), 2.29 (t, 2H, *J*_2′,3′_ = 7.5 Hz, H-2′), 1.61 (tt, 2H, *J*_19,18_ = 6.7 Hz, H-19), 1.61 (tt, 2H, *J*_3′,4′_ = 6.7 Hz, H-3′), 1.57 (tt, 2H, *J*_2,3_ = 7.5 Hz, H-2), 1.38–1.20 (m, 56H, H-3–H-18, H-4′–H-15′) and 0.88 (t, 3H, *J*_16′,15′_ = 6.8 Hz, H-16′) ppm.

^13^C NMR (125.68 MHz, 25 °C, CDCl_3_): δ 174.2 (C-1′), 64.6 (C-20), 63.3 (C-1), 34.6 (C-2′), 33.0 (C-2), 32.1 (C-14′), 29.8–29.3 (C-4–C-17, C-4′–C-13′), 28.8 (C-19), 26.1 (C-18), 25.9 (C-3), 25.2 (C-3′), 22.8 (C-15′) and 14.3 (C-16′) ppm.

HRMS (EI): *m*/*z* calcd for C_36_H_72_O_3_Na [M + Na]^+^, 575.5374; found, 575.5346.

#### 20-(Palmitoleoyloxy)eicosan-1-ol (**6**)

Compound **6** was synthesized according to the general procedure for synthesis of OAHFAls. The following amounts of the reagents were used: palmitoleic acid (214.7 mg, 0.84 mmol, 0.9 equiv), NaHSO_4_·H_2_O (4.5 mg, 0.03 mmol, 3.5 mol %), and 1,20-eicosanediol (295.0 mg, 0.94 mmol, 1.0 equiv). The reaction temperature was 90 °C and the reaction time 3 h. A white solid was received after purification (187.4 mg, 40%). *R*_*f*_ = 0.40 (*n*-hexane/EtOAc 4:1), mp 57.0 °C.

^1^H NMR (499.82 MHz, 25 °C, CDCl_3_): δ 5.35 (dtt, 1H, *J*_9′,11′_ = −1.7, *J*_9′,8′_ = 7.3, *J*_9′,10′_ = 10.8 Hz, H-9′), 5.34 (dtt, 1H, *J*_10′,8′_ = −1.7, *J*_10′,11′_ = 7.3 Hz, H-10′), 4.05 (t, 2H, *J*_20,19_ = 6.8 Hz, H-20), 3.64 (t, 2H, *J*_1,2_ = 6.7 Hz, H-1), 2.29 (t, 2H, *J*_2′,3′_ = 7.6 Hz, H-2′), 2.01 (ddt, 2H, *J*_8′,7′_ = 6.7 Hz, H-8′), 2.01 (ddt, 2H, *J*_11′,12′_ = 6.8 Hz, H-11′), 1.62 (tt, 2H, *J*_19,18_ = 6.7 Hz, H-19), 1.60 (tt, 2H, *J*_3′,4′_ = 7.1 Hz, H-3′), 1.57 (tt, 2H, *J*_2,3_ = 6.8 Hz, H-2), 1.38–1.20 (m, 48H, H-3–H-18, H-4′–H-7′, H-12′–H-15′) and 0.88 (t, 3H, *J*_16′,15′_ = 6.7 Hz, H-16′) ppm.

^13^C NMR (125.68 MHz, 25 °C, CDCl_3_): δ 174.1 (C-1′), 130.1 (C-9′), 129.9 (C-10′), 64.5 (C-20), 63.2 (C-1), 34.5 (C-2′), 33.0 (C-2), 31.9 (C-14′), 29.9–29.2 (C-4–C-17, C-4′–C-7′, C-12′–C-13′), 28.8 (C-19), 27.4–27.3 (C-8′, C-11′), 26.1 (C-18), 25.9 (C-3), 25.2 (C-3′), 22.8 (C-15′) and 14.2 (C-16′) ppm.

HRMS (EI): *m*/*z* calcd for C_36_H_70_O_3_Na [M + Na]^+^, 573.5217; found, 573.5171.

#### 20-(Stearoyloxy)eicosan-1-ol (**7**)

Compound **7** was synthesized according to the general procedure for synthesis of OAHFAls. The following amounts of the reagents were used: stearic acid (185.4 mg, 0.65 mmol, 1.0 equiv), NaHSO_4_·H_2_O (3.2 mg, 0.02 mmol, 3.5 mol %), and 1,20-eicosanediol (211.1 mg, 0.67 mmol, 1.0 equiv). The reaction temperature was 95 °C and the reaction time 4 h. A white solid was received after purification (124.0 mg, 32%). *R*_*f*_ = 0.46 (*n*-hexane/EtOAc 4:1), mp 77.9 °C.

^1^H NMR (499.82 MHz, 25 °C, CDCl_3_): δ 4.05 (t, 2H, *J*_20,19_ = 6.8 Hz, H-20), 3.64 (dt, 2H, *J*_1,1-OH_ = 5.4 Hz, *J*_1,2_ = 6.6 Hz, H-1), 2.29 (t, 2H, *J*_2′,3′_ = 7.5 Hz, H-2′), 1.61 (tt, 2H, *J*_19,18_ = 6.7 Hz, H-19), 1.61 (tt, 2H, *J*_3′,4′_ = 6.7 Hz, H-3′), 1.57 (tt, 2H, *J*_2,3_ = 7.6 Hz, H-2), 1.38–1.21 (m, 60H, H-3–H-18, H-4′–H-17′), 1.20 (t, 1H, 1-OH) and 0.88 (t, 3H, *J*_18′,17′_ = 7.2 Hz, H-18′) ppm.

^13^C NMR (125.68 MHz, 25 °C, CDCl_3_): δ 174.2 (C-1′), 64.6 (C-20), 63.3 (C-1), 34.6 (C-2′), 33.0 (C-2), 32.1 (C-16′), 29.9–29.3 (C-4–C-17, C-4′–C-15′), 28.8 (C-19), 26.1 (C-18), 25.9 (C-3), 25.2 (C-3′), 22.9 (C-17′) and 14.3 (C-18′) ppm.

HRMS (EI): *m*/*z* calcd for C_38_H_77_O_3_ [M + H]^+^, 581.5867; found, 581.5679.

#### 20-(Linoleoyloxy)eicosan-1-ol (**8**)

Compound **8** was synthesized according to the general procedure for synthesis of OAHFAls. The following amounts of the reagents were used: linoleic acid (262.0 mg, 0.93 mmol, 1.0 equiv), NaHSO_4_·H_2_O (4.8 mg, 0.03 mmol, 3.5 mol %), and 1,20-eicosanediol (309.2 mg, 0.98 mmol, 1.0 equiv). The reaction temperature was 95 °C and the reaction time 5 h. A white solid was received after purification (183.6 mg, 32%). *R*_*f*_ = 0.47 (*n*-hexane/EtOAc 4:1), mp 51.0 °C.

^1^H NMR (499.82 MHz, 25 °C, CDCl_3_): δ 5.38 (dtt, 1H, *J*_9′,11′_ = −1.9, *J*_9′,8′_ = 7.2, *J*_9′,10′_ = 10.7 Hz, H-9′), 5.37 (dtt, 1H, *J*_13′,11′_ = −1.6, *J*_13′,14′_ = 7.1, *J*_13′,12′_ = 10.7 Hz, H-13′), 5.33 (dtt, 1H, *J*_10′,8′_ = −1.9, *J*_10′,11′_ = 6.8 Hz, H-10′), 5.33 (dtt, 1H, *J*_12′,14′_ = −2.1, *J*_12′,11′_ = 7.7 Hz, H-12′), 4.05 (t, 2H, *J*_20,19_ = 6.8 Hz, H-20), 3.64 (dt, 2H, *J*_1,1-OH_ = 5.4, *J*_1,2_ = 6.7 Hz, H-1), 2.77 (dddd, 2H, H-11′), 2.28 (t, 2H, *J*_2′,3′_ = 7.6 Hz, H-2′), 2.05 (ddt, 2H, *J*_8′,7′_ = 7.3 Hz, H-8′), 2.05 (ddt, 2H, *J*_14′,15′_ = 6.9 Hz, H-14′), 1.62 (tt, 2H, *J*_19,18_ = 6.7 Hz, H-19), 1.61 (tt, 2H, *J*_3′,4′_ = 6.9 Hz, H-3′), 1.56 (tt, 2H, *J*_2,3_ = 6.9 Hz, H-2), 1.39–1.22 (m, 46H, H-3–H-18, H-4′–H-7′, H-15′–H-17′), 1.22 (t, 1H, 1-OH) and 0.88 (t, 3H, *J*_18′,17′_ = 6.7 Hz, H-18′) ppm.

^13^C NMR (125.68 MHz, 25 °C, CDCl_3_): δ 174.1 (C-1′), 130.4 (C-9′), 130.2 (C-13′), 128.2 (C-10′), 128.1 (C-12′), 64.6 (C-20), 63.2 (C-1), 34.6 (C-2′), 33.0 (C-2), 31.7 (C-16′), 29.8–29.3 (C-4–C-17, C-4′–C-7′, C-15′), 28.8 (C-19), 27.3 (C-8′, C-14′), 26.1 (C-18), 25.9 (C-3), 25.2 (C-11′), 24.9 (C-3′), 22.7 (C-17′) and 14.2 (C-18′) ppm.

HRMS (EI): *m*/*z* calcd for C_38_H_72_O_3_Na [M + Na]^+^, 599.5374; found, 599.5357.

#### 12-(Palmitoyloxy)dodecanoic Acid (**9**)

Compound **9** was synthesized according to the general procedure for synthesis of OAHFAs. The following amounts of the reagents were used: **1** (72.6 mg, 0.16 mmol, 1.0 equiv) and Jones’ reagent (272 μL, 0.54 mmol, 3.3 equiv). The reaction time was 4 h. A white solid was received after purification (68.2 mg, 91%). *R*_*f*_ = 0.37 (*n*-hexane/EtOAc/AcOH 7:3:0.01), mp 66.7 °C.

^1^H NMR (499.82 MHz, 25 °C, CDCl_3_): δ 4.05 (t, 2H, *J*_12,11_ = 6.7 Hz, H-12), 2.35 (t, 2H, *J*_2,3_ = 7.5 Hz, H-2), 2.29 (t, 2H, *J*_2′,3′_ = 7.5 Hz, H-2′), 1.63 (tt, 2H, *J*_3,4_ = 6.7 Hz, H-3), 1.61 (tt, 2H, *J*_11,10_ = 6.7 Hz, H-11), 1.61 (tt, 2H, *J*_3′,4′_ = 6.8 Hz, H-3′), 1.37–1.22 (m, 38H, H-4–H-10, H-4′–H-15′) and 0.88 (t, 3H, *J*_16′,15′_ = 6.8 Hz, H-16′) ppm.

^13^C NMR (125.68 MHz, 25 °C, CDCl_3_): δ 179.2 (C-1), 174.2 (C- 1′), 64.6 (C-12), 34.6 (C-2′), 34.0 (C-2), 32.1 (C-14′), 29.8–29.2 (C-4–C-9, C-4′–C-13′), 28.8 (C-11), 26.1 (C-10), 25.2 (C-3′), 24.8 (C-3), 22.8 (C-15′) and 14.3 (C-16′) ppm.

HRMS (EI): *m*/*z* calcd for C_28_H_54_O_4_Na [M + Na]^+^, 477.3914; found, 477.3868.

#### 12-(Palmitoleoyloxy)dodecanoic Acid (**10**)

Compound **10** was synthesized according to the general procedure for synthesis of OAHFAs. The following amounts of the reagents were used: **2** (35.8 mg, 0.08 mmol, 1.0 equiv.) and Jones’ reagent (94 μL, 0.19 mmol, 2.3 equiv.). The reaction time was 2.5 h. A white solid was received after purification (62.3 mg, 92%). *R*_*f*_ = 0.43 (*n*-hexane/EtOAc/AcOH 7:3:0.01), mp 36.8 °C.

^1^H NMR (499.82 MHz, 25 °C, CDCl_3_): δ 5.35 (dtt, 1H, *J*_9′,11′_ = −1.9, *J*_9′,8′_ = 7.3, *J*_9′,10′_ = 10.3 Hz, H-9′), 5.34 (dtt, 1H, *J*_10′,8′_ = −2.0, *J*_10′,11′_ = 7.2 Hz, H-10′), 4.05 (t, 2H, *J*_12,11_ = 6.7 Hz, H-12), 2.35 (t, 2H, *J*_2,3_ = 7.2 Hz, H-2), 2.29 (t, 2H, *J*_2′,3′_ = 7.6 Hz, H-2′), 2.01 (ddt, 2H, *J*_8′,7′_ = 6.9 Hz, H-8′), 2.01 (ddt, 2H, *J*_11′,12′_ = 6.7 Hz, H-11′), 1.63 (tt, 2H, *J*_3,4_ = 6.9 Hz, H-3), 1.62 (tt, 2H, *J*_11,10_ = 7.1 Hz, H-11), 1.61 (tt, 2H, *J*_3′,4′_ = 6.8 Hz, H-3′), 1.40–1.20 (m, 30H, H-4–H-10, H-4′–H-7′, H-12′–H-15′) and 0.88 (t, 3H, *J*_16′,15′_ = 6.7 Hz, H-16′) ppm.

^13^C NMR (125.68 MHz, 25 °C, CDCl_3_): δ 179.2 (C-1), 174.2 (C- 1′), 130.1 (C-9′), 129.9 (C-10′), 64.6 (C-12), 34.6 (C-2′), 34.0 (C-2), 31.9 (C-14′), 29.7–29.0 (C-4–C-9, C-4′–C-7′, C-12′– C-13′), 28.8 (C-11), 27.4–27.3 (C-8′, C-11′), 26.1 (C-10), 25.2 (C-3′), 24.8 (C-3), 22.9 (C-15′) and 14.2 (C-16′) ppm.

HRMS (EI): *m*/*z* calcd for C_28_H_52_O_4_Na [M + Na]^+^, 475.3758; found, 475.3681.

#### 12-(Stearoyloxy)dodecanoic Acid (**11**)

Compound **11** was synthesized according to the general procedure for synthesis of OAHFAs. The following amounts of the reagents were used: **3** (43.4 mg, 0.09 mmol, 1.0 equiv) and Jones’ reagent (140 μL, 0.28 mmol, 3.0 equiv). The reaction time was 2 h. A white solid was received after purification (32.2 mg, 72%). *R*_*f*_ = 0.42 (*n*-hexane/EtOAc/AcOH 7:3:0.01), mp 71.2 °C.

^1^H NMR (499.82 MHz, 25 °C, CDCl_3_): δ 4.05 (t, 2H, *J*_12,11_ = 6.7 Hz, H-12), 2.35 (t, 2H, *J*_2,3_ = 7.5 Hz, H-2), 2.29 (t, 2H, *J*_2′,3′_ = 7.5 Hz, H-2′), 1.63 (tt, 2H, *J*_3,4_ = 6.7 Hz, H-3), 1.61 (tt, 2H, *J*_3′,4′_ = 6.8 Hz, H-3′), 1.60 (tt, 2H, *J*_11,10_ = 6.7 Hz, H-11), 1.37–1.20 (m, 42H, H-4–H-10, H-4′–H-17′) and 0.88 (t, 3H, *J*_18′,17′_ = 6.8 Hz, H-18′) ppm.

^13^C NMR (125.68 MHz, 25 °C, CDCl_3_): δ 178.9 (C-1), 174.2 (C- 1′), 64.5 (C-12), 34.6 (C-2′), 34.0 (C-2), 32.1 (C-16′), 29.8–29.2 (C-4–C-9, C-4′–C-15′), 28.8 (C-11), 26.1 (C-10), 25.2 (C-3′), 24.8 (C-3), 22.8 (C-17′) and 14.3 (C-18′) ppm.

HRMS (EI): *m*/*z* calcd for C_30_H_58_O_4_Na [M + Na]^+^, 505.4227; found, 505.4251.

#### 12-(Linoleoyloxy)dodecanoic Acid (**12**)

Compound **12** was synthesized according to the general procedure for synthesis of OAHFAs. The following amounts of the reagents were used: **4** (105.2 mg, 0.23 mmol, 1.0 equiv) and Jones’ reagent (250 μL, 0.50 mmol, 2.3 equiv). The reaction time was 2 h. A colorless oil was received after purification (76.4 mg, 71%). *R*_*f*_ = 0.37 (*n*-hexane/EtOAc/AcOH 7:3:0.01), mp 30.3 °C.

^1^H NMR (499.82 MHz, 25 °C, CDCl_3_): δ 5.38 (dtt, 1H, *J*_9′,11′_ = −1.7, *J*_9′,8′_ = 7.2, *J*_9′,10′_ = 10.7 Hz, H-9′), 5.37 (dtt, 1H, *J*_13′,11′_ = −1.6, *J*_13′,14′_ = 7.0, *J*_13′,12′_ = 10.8 Hz, H-13′), 5.33 (dtt, 1H, *J*_10′,8′_ = −1.8, *J*_10′,11′_ = 6.9 Hz, H-10′), 5.33 (dtt, 1H, *J*_12′,14′_ = −2.2, *J*_12′,11′_ = 7.6 Hz, H-12′), 4.05 (t, 2H, *J*_12,11_ = 6.8 Hz, H-12), 2.77 (dddd, 2H, H-11′), 2.35 (t, 2H, *J*_2,3_ = 7.5 Hz, H-2), 2.29 (t, 2H, *J*_2′,3′_ = 7.6 Hz, H-2′), 2.05 (ddt, 2H, *J*_8′,7′_ = 7.3 Hz, H-8′), 2.05 (ddt, 2H, *J*_14′,15′_ = 6.9 Hz, H-14′), 1.63 (tt, 2H, *J*_3,4_ = 7.2 Hz, H-3), 1.61 (tt, 2H, *J*_11,10_ = 7.0 Hz, H-11), 1.61 (tt, 2H, *J*_3′,4′_ = 6.6 Hz, H-3′), 1.39–1.18 (m, 28H, H-3–H-10, H-4′–H-7′, H-15′–H-17′) and 0.88 (t, 3H, *J*_18′,17′_ = 6.9 Hz, H-18′) ppm.

^13^C NMR (125.68 MHz, 25 °C, CDCl_3_): δ 178.9 (C-1), 174.2 (C-1′), 130.4 (C-9′), 130.2 (C-13′), 128.2 (C-10′), 128.1 (C-12′), 64.6 (C-12), 34.6 (C-2′), 33.9 (C-2), 31.7 (C-16′), 29.7–29.2 (C-4–C-9, C-4′–C7′, C-15′), 28.8 (C-11), 27.3 (C-8′, C-14′), 26.1 (C-10), 25.8 (C-11′), 25.2 (C-3′), 24.8 (C-3), 22.7 (C-17′) and 14.2 (C-18′) ppm.

HRMS (EI): *m*/*z* calcd for C_30_H_54_O_4_Na [M + Na]^+^, 501.3914; found, 501.3931.

#### 20-(Palmitoyloxy)eicosanoic Acid (**13**)

Compound **13** was synthesized according to the general procedure for synthesis of OAHFAs. The following amounts of the reagents were used: **5** (22.7 mg, 0.04 mmol, 1.0 equiv) and Jones’ reagent (50 μL, 0.10 mmol, 2.4 equiv). The reaction time was 1.5 h. A white solid was received after purification (19.3 mg, 83%). *R*_*f*_ = 0.57 (*n*-hexane/EtOAc/AcOH 7:3:0.01), mp 76.8 °C.

^1^H NMR (499.82 MHz, 25 °C, CDCl_3_): δ 4.05 (t, 2H, *J*_20,19_ = 6.72 Hz, H-20), 2.35 (t, 2H, *J*_2,3_ = 7.5 Hz, H-2), 2.29 (t, 2H, *J*_2′,3′_ = 7.5 Hz, H-2′), 1.63 (tt, 2H, *J*_3,4_ = 6.9 Hz, H-3), 1.61 (tt, 2H, *J*_19,18_ = 7.5 Hz, H-19), 1.61 (tt, 2H, *J*_3′,4′_ = 6.5 Hz, H-3′), 1.39–1.20 (m, 54H, H-4–H-18, H-4′–H-15′) and 0.88 (t, 3H, *J*_16′,15′_ = 6.95 Hz, H-16′) ppm.

^13^C NMR (125.68 MHz, 25 °C, CDCl_3_): δ 177.5 (C-1), 174.2 (C-1′), 64.6 (C-20), 34.6 (C-2′), 33.9 (C-2), 32.1 (C-14′), 29.8–29.1 (C-4–C-17, C-4′–C-13′), 28.8 (C-19), 26.1 (C-18), 25.2 (C-3′), 24.9 (C-3), 22.8 (C-15′) and 14.3 (C-16′) ppm.

HRMS (EI): *m*/*z* calcd for C_36_H_70_O_4_Na [M + Na]^+^, 589.5166; found, 589.5145.

#### 20-(Palmitoleoyloxy)eicosanoic Acid (**14**)

Compound **14** was synthesized according to the general procedure for synthesis of OAHFAs. The following amounts of the reagents were used: **6** (39.6 mg, 0.07 mmol, 1.0 equiv) and Jones’ reagent (130 μL, 0.26 mmol, 3.6 equiv). The reaction time was 3 h. A white solid was received after purification (36.3 mg, 89%). *R*_*f*_ = 0.54 (*n*-hexane/EtOAc/AcOH 7:3:0.01), mp 61.0 °C.

^1^H NMR (499.82 MHz, 25 °C, CDCl_3_): δ 5.35 (dtt, 1H, *J*_9′,11′_ = −1.6, *J*_9′,8′_ = 7.3, *J*_9′,10′_ = 10.9 Hz, H-9′), 5.34 (dtt, 1H, *J*_10′,8′_ = −1.7, *J*_10′,11′_ = 7.2 Hz, H-10′), 4.05 (t, 2H, *J*_20,19_ = 6.8 Hz, H-20), 2.35 (t, 2H, *J*_2,3_ = 7.5 Hz, H-2), 2.29 (t, 2H, *J*_2′,3′_ = 7.5 Hz, H-2′), 2.01 (ddt, 2H, *J*_8′,7′_ = 6.9 Hz, H-8′), 2.01 (ddt, 2H, *J*_11′,12′_ = 6.7 Hz, H-11′), 1.63 (tt, 2H, *J*_3,4_ = 6.9 Hz, H-3), 1.61 (tt, 2H, *J*_19,18_ = 7.5 Hz, H-19), 1.61 (tt, 2H, *J*_3′,4′_ = 6.5 Hz, H-3′), 1.40–1.20 (m, 46H, H-4–H-18, H-4′–H-7′, H-12′–H-15′) and 0.88 (t, 3H, *J*_16′,15′_ = 6.7 Hz, H-16′) ppm.

^13^C NMR (125.68 MHz, 25 °C, CDCl_3_): δ 178.6 (C-1), 174.2 (C-1′), 130.1 (C-9′), 129.9 (C-10′), 64.6 (C-20), 34.6 (C-2′), 33.9 (C-2), 31.9 (C-14′), 29.9–29.1 (C-4–C-17, C-4′–C-7′, C-12′–C-13′), 28.8 (C-19), 27.4–27.3 (C-8′, C-11′), 26.1 (C-18), 25.2 (C-3′), 24.9 (C-3), 22.8 (C-15′) and 14.2 (C-16′) ppm.

HRMS (EI): *m*/*z* calcd for C_36_H_68_O_4_Na [M + Na]^+^, 587.5010; found, 587.5051.

#### 20-(Stearoyloxy)eicosanoic Acid (**15**)

Compound **15** was synthesized according to the general procedure for synthesis of OAHFAs. The following amounts of the reagents were used: **7** (43.4 mg, 0.07 mmol, 1.0 equiv) and Jones’ reagent (120 μL, 0.24 mmol, 3.2 equiv). The reaction time was 1.5 h. A white solid was received after purification (32.2 mg, 72%). *R*_*f*_ = 0.59 (*n*-hexane/EtOAc/AcOH 7:3:0.01), mp 81.3 °C.

^1^H NMR (499.82 MHz, 25 °C, CDCl_3_): δ 4.05 (t, 2H, *J*_20,19_ = 6.7 Hz, H-20), 2.35 (t, 2H, *J*_2,3_ = 7.5 Hz, H-2), 2.29 (t, 2H, *J*_2′,3′_ = 7.6 Hz, H-2′), 1.63 (tt, 2H, *J*_3,4_ = 6.7 Hz, H-3), 1.61 (tt, 2H, *J*_3′,4′_ = 6.8 Hz, H-3′), 1.61 (tt, *J*_19,18_ = 7.6 Hz, 2H, H-19), 1.39–1.20 (m, 58H, H-4–H-18, H-4′–H-17′) and 0.88 (t, *J*_18′,17′_ = 7.0 Hz, 3H, H-18′) ppm.

^13^C NMR (125.68 MHz, 25 °C, CDCl_3_): δ 179.1 (C-1), 174.2 (C-1′), 64.6 (C-20), 34.6 (C-2′), 34.0 (C-2), 32.1 (C-16′), 29.9–29.2 (C-4–C-17, C-4′–C-15′), 28.8 (C-19), 26.1 (C-18), 25.2 (C-3′), 24.9 (C-3), 22.9 (C-17′) and 14.3 (C-18′) ppm.

HRMS (EI): *m*/*z* calcd for C_38_H_75_O_4_ [M + H]^+^, 595.5660; found, 595.5648.

#### 20-(Linoleoyloxy)eicosanoic Acid (**16**)

Compound **16** was synthesized according to the general procedure for synthesis of OAHFAs. The following amounts of the reagents were used: **8** (87.5 mg, 0.15 mmol, 1.0 equiv) and Jones’ reagent (174 μL, 0.35 mmol, 2.3 equiv). The reaction time was 2 h. A white solid was received after purification (74.7 mg, 83%). *R*_*f*_ = 0.63 (*n*-hexane/EtOAc/AcOH 7:3:0.01), mp 53.6 °C.

^1^H NMR (499.82 MHz, 25 °C, CDCl_3_): δ 5.38 (dtt, 1H, *J*_9′,11′_ = −1.8, *J*_9′,8′_ = 6.8, *J*_9′,10′_ = 10.7 Hz, H-9′), 5.37 (dtt, 1H, *J*_13′,11′_ = −1.8, *J*_13′,14′_ = 6.6, *J*_13′,12′_ = 10.8 Hz, H-13′), 5.33 (dtt, 1H, *J*_10′,8′_ = −1.8, *J*_10′,11′_ = 6.9 Hz, H-10′), 5.33 (dtt, 1H, *J*_12′,14′_ = −2.1, *J*_12′,11′_ = 7.6 Hz, H-12′), 4.05 (t, 2H, *J*_20,19_ = 6.8 Hz, H-20), 2.77 (dddd, 2H, H-11′), 2.35 (t, 2H, *J*_2,3_ = 7.5 Hz, H-2), 2.29 (t, 2H, *J*_2′,3′_ = 7.5 Hz, H-2′), 2.05 (ddt, 2H, *J*_8′,7′_ = 7.4 Hz, H-8′), 2.05 (ddt, 2H, *J*_14′,15′_ = 6.7 Hz, H-14′), 1.63 (tt, 2H, *J*_3,4_ = 6.9 Hz, H-3), 1.61 (tt, 2H, *J*_19,18_ = 7.1 Hz, H-19), 1.60 (tt, 2H, *J*_3′,4′_ = 6.6 Hz, H-3′), 1.39–1.18 (m, 44H, H-3–H-18, H-4′–H-7′, H-15′–H-17′) and 0.89 (t, 3H, *J*_18′,17′_ = 6.9 Hz, H-18′) ppm.

^13^C NMR (125.68 MHz, 25 °C, CDCl_3_): δ 178.6 (C-1), 174.2 (C-1′), 130.4 (C-9′), 130.2 (C-13′), 128.2 (C-10′), 128.1 (C-12′), 64.6 (C-20), 34.6 (C-2′), 33.9 (C-2), 31.7 (C-16′), 29.8–29.2 (C-4–C-17, C-4′–C-7′, C-15′), 28.8 (C-19), 27.4 (C-8′, C-14′), 26.1 (C-18), 25.8 (C-11′), 25.2 (C-3′), 24.9 (C-3), 22.7 (C-17′) and 14.2 (C-18′) ppm.

HRMS (EI): *m*/*z* calcd for C_38_H_70_O_4_Na [M + Na]^+^, 613.5166; found, 613.5164.

### Langmuir Monolayer Studies

#### General Instrumental Details

The Langmuir trough studies were performed with a KSV large Langmuir trough (Biolin Scientific, Espoo, Finland; dimensions 580 × 145 mm). A PBS-buffer (140 mM NaCl, 3 mM KCl, 10 mM phosphate buffer, pH 7.4) was used as a subphase. The temperature was kept at the ocular surface temperature of 35 ± 1 °C during the experiments and the temperature was controlled using a circulating water bath (LAUDA ECO E4, Germany). The entire measurement setup was enclosed in an acrylic box (volume 250 L) and dry air was continuously led into the container at a rate of 76 L/min through an ozone solutions ODS-3P ozone destruct unit (Hull, Iowa) to maintain a low-ozone atm and prevent the potential oxidation of the lipid species during experiments.

#### Surface Pressure/Surface Potential Measurements and BAM-Imaging

The surface pressure and surface potential measurements, as well as BAM-imaging, were performed in the following way: the lipid samples (5 mM chloroform solutions) were added onto the PBS subphase using a 50 μL Hamilton syringe (Hamilton Central Europe S.R.L., Ghiroda, Romania) or a 50 μL eVol XR digital analytical syringe (SGE Analytical Science, Ringwood, VIC, Australia). The chloroform was allowed to evaporate for 5 min after which the film was compressed with a barrier speed of 10 mm/min. The surface pressure was measured using a Wilhelmy plate and the surface potential was measured with a KSV surface potential sensor (Espoo, Finland). BAM-imaging was performed with a KSV NIMA microBAM camera (Espoo, Finland). The respreading capabilities of the lipid films were assessed by measuring the surface pressure changes over five compression–expansion cycles (barrier speed 250 mm/min). All measurements were performed in triplicates.

The effective molecular dipole moment was calculated with eq 1, where Δ*V* is the measured surface potential, *A* is the mean molecular area, and ε_0_ is the permittivity of free space.
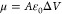
1

#### Oscillating Barrier Experiment

In addition to these measurements, the viscoelastic properties were also studied. The viscoelastic properties of the lipids were studied through an oscillating barrier experiment over the frequency range 100–250 mHz at a set surface pressure of 30 mN/m. A more detailed description can be found in the literature.^36^

#### Evaporation Resistance

The evaporation resistance of a lipid film was determined in the following way: the lipids were added onto the PBS subphase of the KSV large Langmuir trough, the chloroform was allowed to evaporate for 5 min, and then the film was compressed to a set area/molecule (ranging between 30 and 5 Å^2^). The evaporation resistance was then measured at these areas using a modified version of the Langmuir–Schaefer method.^39^ A desiccant container filled with silica gel was placed onto a holder a few millimeters above the subphase surface and kept in place for 5 min. The desiccant containers used in the measurements were disposable desiccant cartridges (SP Industries, Warminster, PA) covered with Millipore Immobilon -P PVDF membrane with 450 nm pore size (Bedford, MA). After the experiment, the container was weighed to determine how much the lipid film reduced evaporation. A reference measurement was also done to the subphase as such, and thus the evaporation resistance of the lipid film could be calculated using eq 2.
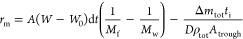
2

## Conclusions

The TFLL OAHFAs have emerged as an interesting class of molecules which play an essential role in the amphiphilic lipid layer residing at the interface between the aqueous tear film and the thicker nonpolar lipid layer. Previous work in the field has focused on the synthesis and profiling of OAHFA species bearing a C_18:1_ acyl group. Herein, we set out to study the properties of other OAHFA species bearing either a C_16:0_, C_16:1_, C_18:0_ or C_18:2_ acyl group to complement the existing literature data as well as identify trends which can be utilized to characterize these species or predict their biophysical properties.

We started the study by synthesizing and characterizing a representative library of OAHFAls and OAHFAs bearing two variations in the parent chain and four variations in the acyl group. After a detailed NMR-spectroscopic characterization of the pure species, we sought to identify specific patterns in the ^1^H and ^13^C NMR spectra which could be utilized as part of an assessment platform of more complex OAHFA-mixtures. The analytical data generated will be of importance to chemistry teams focusing on the characterization of synthetic lipid species, but we do not foresee that NMR spectroscopic techniques will be able to outcompete the sensitivity and accuracy of mass spectrometry-based tools in more demanding characterization studies in the near future.

Determination of the melting points of the OAHFAl and OAHFA libraries yielded detailed insights on the effects of terminal functional group, degree of saturation, and chain length on the melting points of these lipid classes. The general trends identified could be verified by analyzing the melting points of previously reported TFLL OAHFAs and their structural analogues. As a result of our studies, a sound foundation which can be utilized for accurate prediction of melting points in distinct OAHFAs is now available.

The Langmuir monolayer studies performed were intended to shed light on the correlation between melting points and film behavior, factors that have previously been considered important for unravelling the mysteries of the TFLL. Our studies indicate that the correlation between melting points and film behavior is not clear-cut. Nevertheless, we were able to group the OAHFA species into three categories based on their melting points which display distinct behavior at the aqueous interface under ocular surface temperature and pressure. While the different acyl groups inflict a change in the fundamental biophysical properties of the OAHFAs, our results indicate that the structure of the entire molecule including both the parent chain and the acyl group needs to be assessed together as a single entity.

Lastly, in addition to providing multidisciplinary new insights on the properties of TFLL OAHFAs and a solid base for future assessment of such species, the results from the structure–property profiling studies featuring a library of lipids are expected to be of interest to the surface science community. The questions which we were not able to fully address in the current study will require access to a larger lipid library with additional structural variations and we will be returning to this topic as part of future work.

## Supporting Information Available

^1^H and ^13^C NMR spectra for all reported compounds and Supporting Information related to the biophysical studies (PDF)
