# Supplementary material for: Decipher ‘Em All: A Profiling Study on the Effects of Acyl Groups in O-Acyl-ω-hydroxy Fatty Acids
Source: Langmuir. 2024 Oct 3;40(41):21559–72. doi: 10.1021/acs.langmuir.4c02469 (PMC11483765; doi:10.1021/acs.langmuir.4c02469)
Supplement: Supplementary file 1 — la4c02469_si_001.pdf [file la4c02469_si_001.pdf]

Supporting information

Decipher 'Em All: A Profiling Study on the Effects of Acyl Groups in *O*-Acyl-ω-Hydroxy Fatty Acids

Henrik Stubb,[1,ψ] Julia Sevón,[1,ψ] Cordula Schlegel,[1,ψ] Mira Viljanen,[1,2] Jukka Moilanen,[2] Tuomo Viitaja[*,1,2] and Filip S. Ekholm[*,1]

[1] Department of Chemistry, University of Helsinki, P.O. Box 55, FI-00014 Helsinki, Finland
[2] Ophthalmology, University of Helsinki and Helsinki University Hospital, Haartmaninkatu 8, FI-00290 Helsinki, Finland

[ψ] Equal contributions

***Corresponding author contact:** tuomo.viitaja@helsinki.fi, filip.ekholm@helsinki.fi

Contents

1. Examples of viscoelastic measurements in the studied OAHFAs……………………………..…S3

2. Surface pressure isotherms over compression expansion cycles…………………………..……..…S4

3. ^{1}H and ^{13}C NMR spectra of synthesized compounds…………………………………………….…….S5

1. Examples of viscoelastic measurements in the studied OAHFAs

Tabulated values for the storage modulus (G’), the loss modulus (G’’) as well as the calculated $\tan(\delta)$ can be found below.

$$\textbf{Equation used for calculating } \tan(\boldsymbol{\delta})\textbf{:}\ \tan(\delta) = \frac{G''}{G'}$$

Table S1. The storage modulus (G’), the loss modulus (G’’) and the calculated $\tan(\delta)$ for OAHFAs **9**–**12** (**9:** $C_{12:0}/C_{16:0}$, **10:** $C_{12:0}/C_{16:1}$, **11:** $C_{12:0}/C_{18:0}$, **12:** $C_{12:0}/C_{18:2}$).

	**12:0/18:2-OAHFA**			**12:0/18:0-OAHFA**			**12:0/16:1-OAHFA**			**12:0/16:0-OAHFA**		
freq. (mHz)	G'	G''	$\tan(\delta)$	G'	G''	$\tan(\delta)$	G'	G''	$\tan(\delta)$	G'	G''	$\tan(\delta)$
100	26,89	14,13	0,53	126,5	56,19	0,44	28,87	15,14	0,52	114,1	75,72	0,66
150	26,67	15,84	0,59	124,6	66,74	0,54	27,69	17,28	0,62	109,8	77,04	0,70
200	25,09	17,05	0,68	126,5	75,88	0,60	27,12	17,70	0,65	107,1	85,53	0,80
250	23,87	18,86	0,79	120,9	82,10	0,68	26,06	19,03	0,73	100,8	87,16	0,86

Table S2. The storage modulus (G’), the loss modulus (G’’) and the calculated $\tan(\delta)$ for OAHFAs **13**–**16** (**13:** $C_{20:0}/C_{16:0}$, **14:** $C_{20:0}/C_{16:1}$, **15:** $C_{20:0}/C_{18:0}$, **16:** $C_{20:0}/C_{18:2}$).

	**20:0/18:2-OAHFA**			**20:0/18:0-OAHFA**			**20:0/16:1-OAHFA**			**20:0/16:0-OAHFA**		
freq. (mHz)	G'	G''	$\tan(\delta)$	G'	G''	$\tan(\delta)$	G'	G''	$\tan(\delta)$	G'	G''	$\tan(\delta)$
100	104,8	51,68	0,49	431,4	138,5	0,32	190,5	84,12	0,44	184,9	81,48	0,44
150	113,5	73,18	0,64	420,0	162,9	0,39	177,6	83,34	0,47	187,2	97,16	0,52
200	100,3	66,43	0,66	409,7	180,4	0,44	172,6	90,14	0,52	187,1	112,7	0,60
250	116,3	92,53	0,80	404,6	194,6	0,48	168,5	108,4	0,64	174,8	108,8	0,62

2. Surface pressure isotherms over compression expansion cycles

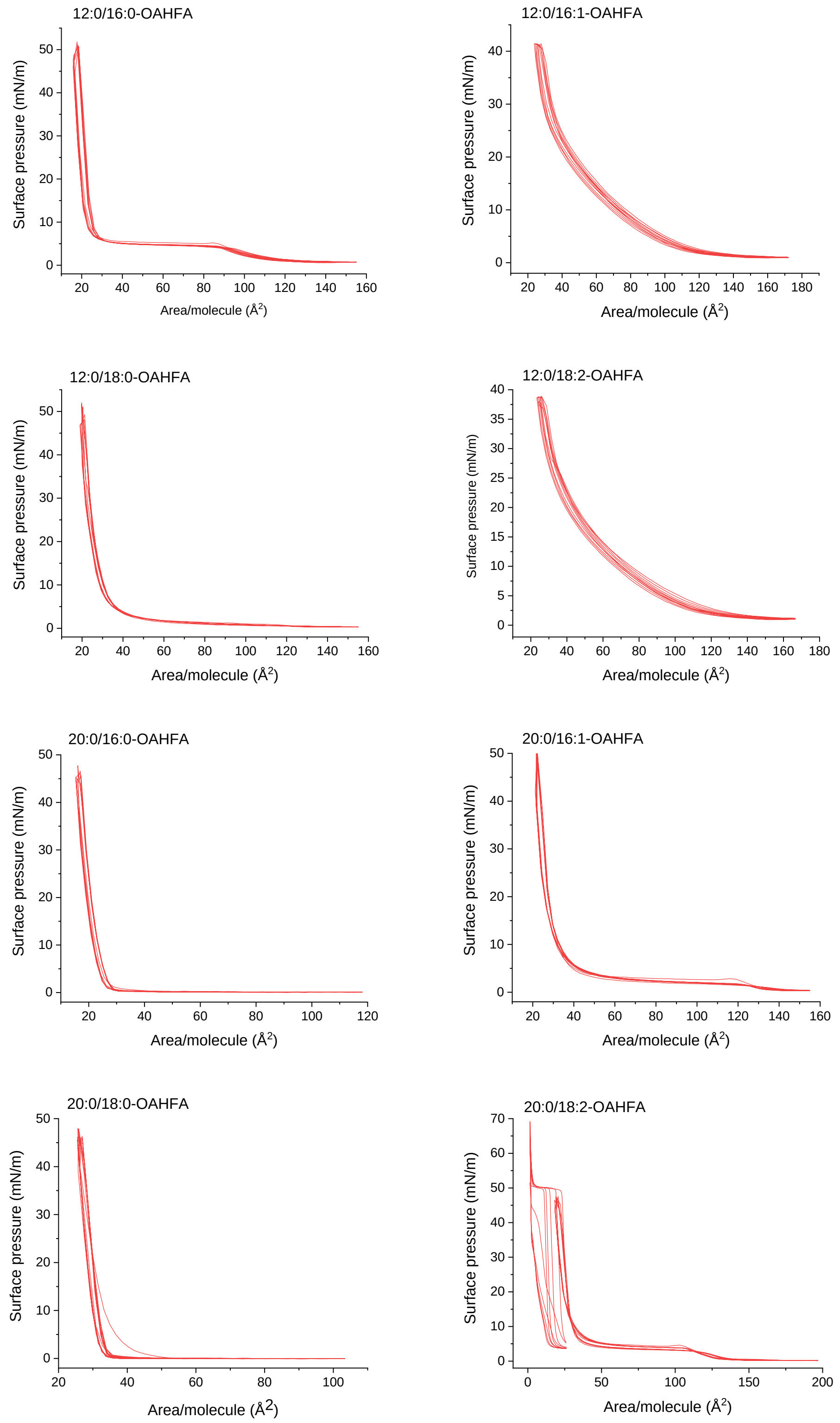

Figure S1. Surface pressure isotherms over compression expansion cycles are showcased as a function of area/molecule. Altogether five compression/expansion cycles were performed.

3. ^1H and ^{13}C-NMR spectra of synthesized compounds

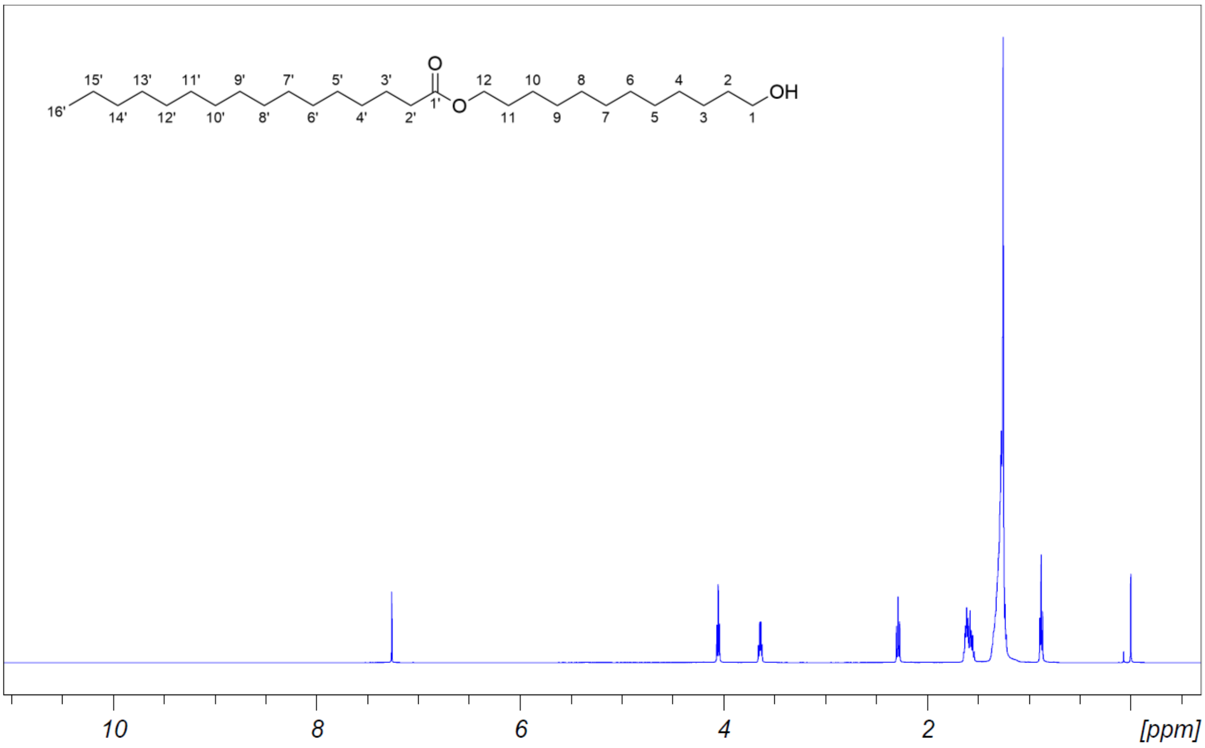

Figure S2. ^1H NMR spectrum of **1** (499.82 MHz, 25 °C).

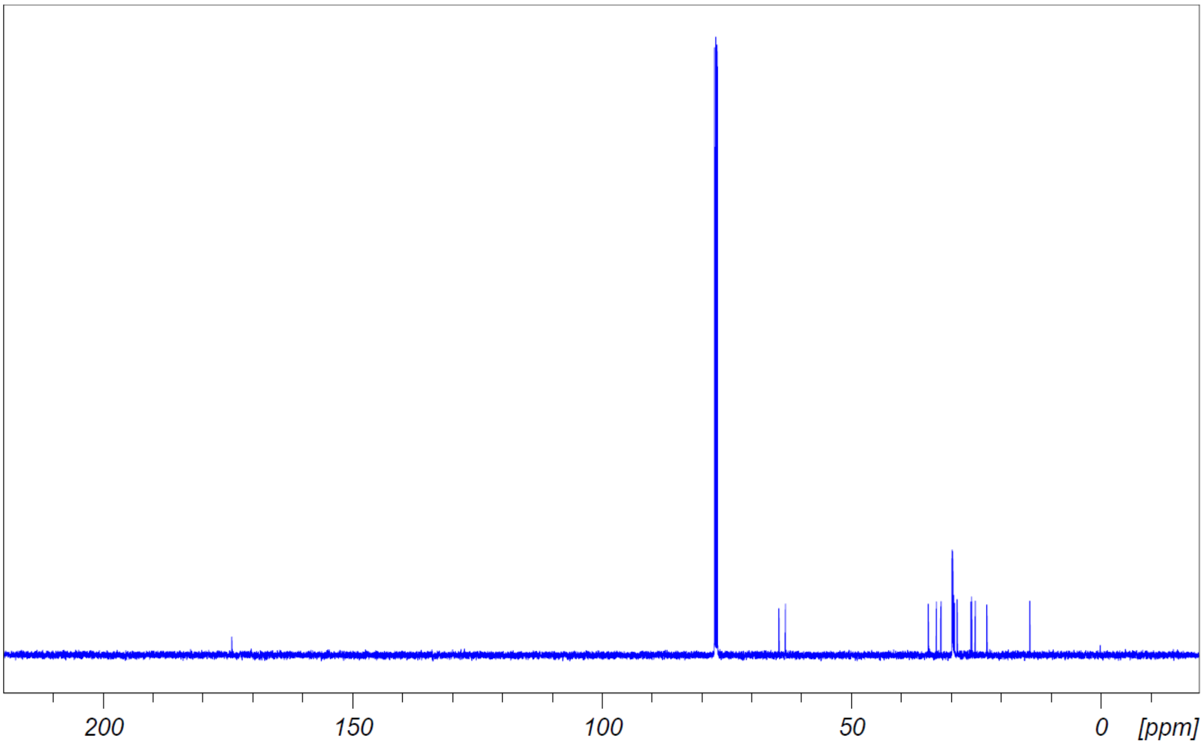

Figure S3. ^{13}C NMR spectrum of **1** (125.68 MHz, 25 °C).

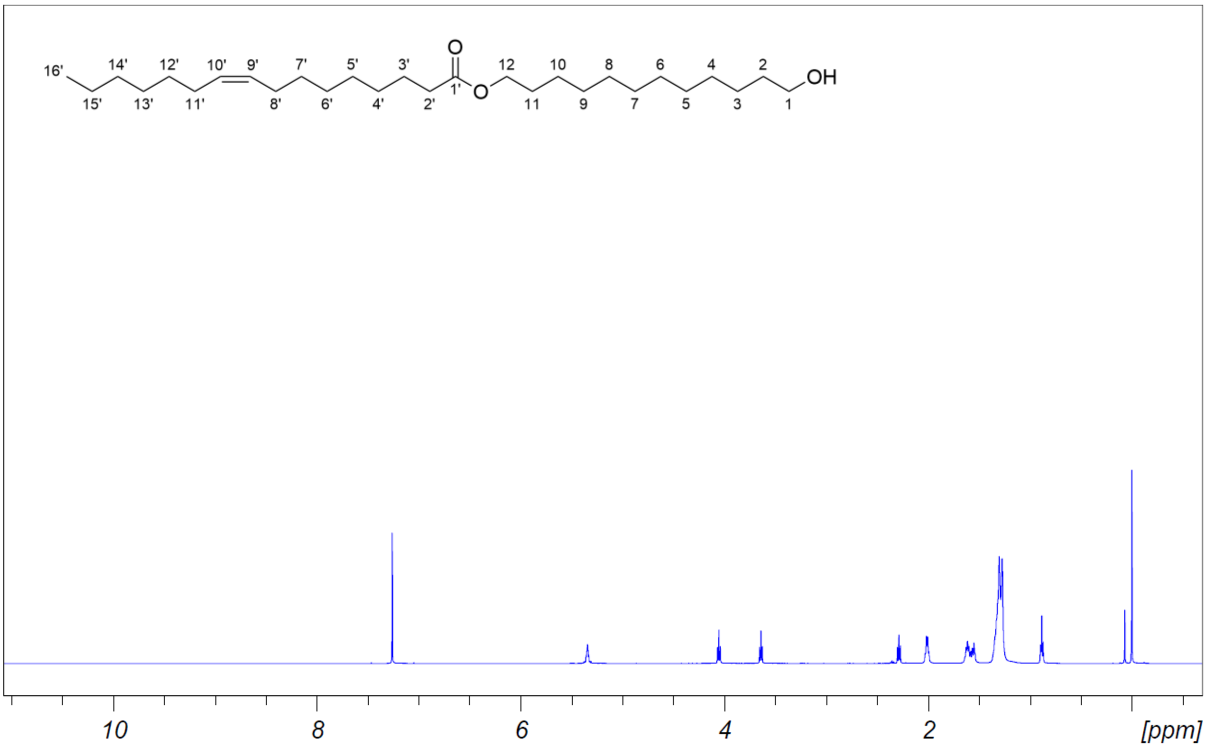

Figure S4. ^{1}H NMR spectrum of **2** (499.82 MHz, 25 °C).

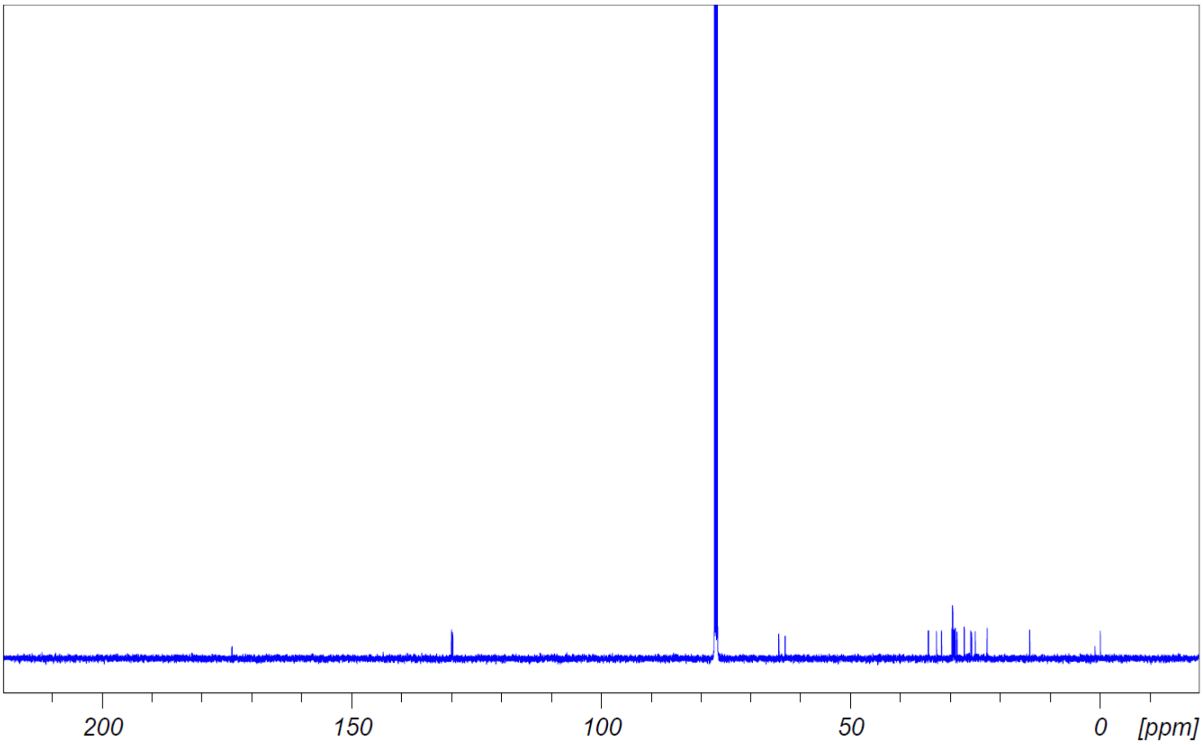

Figure S5. ^{13}C NMR spectrum of **2** (125.68 MHz, 25 °C).

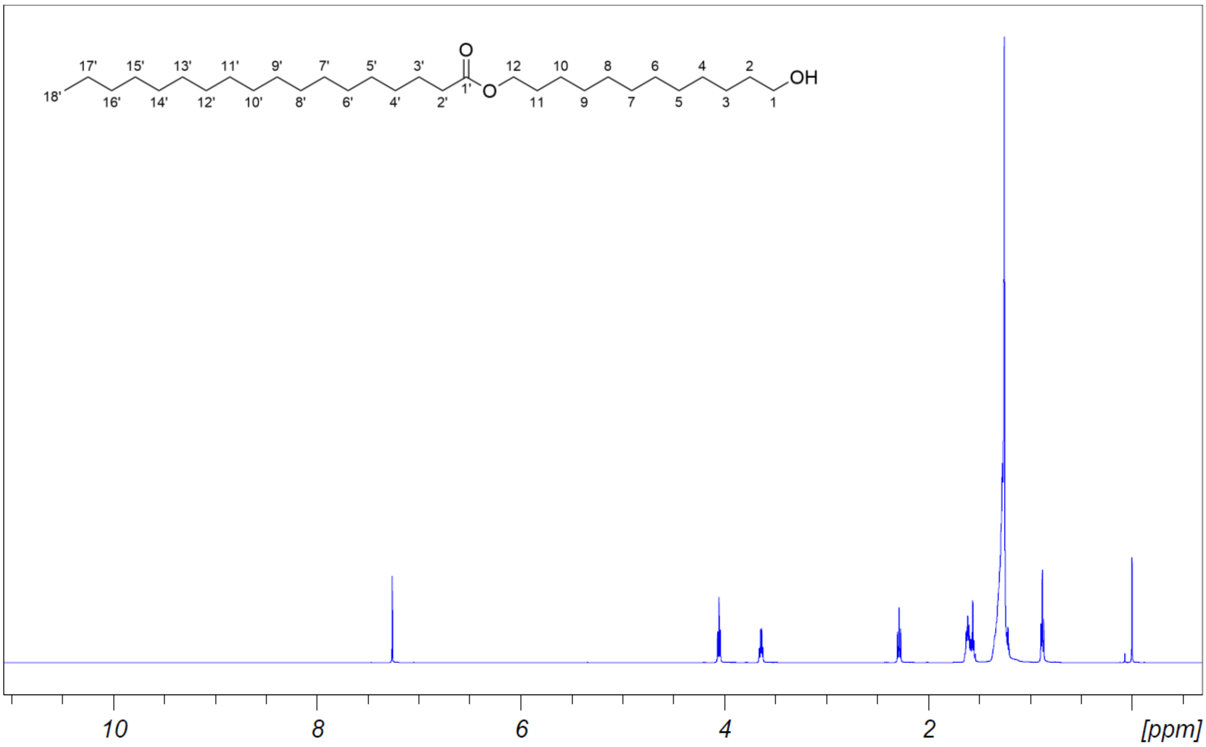

Figure S6. ^{1}H NMR spectrum of **3** (499.82 MHz, 25 °C).

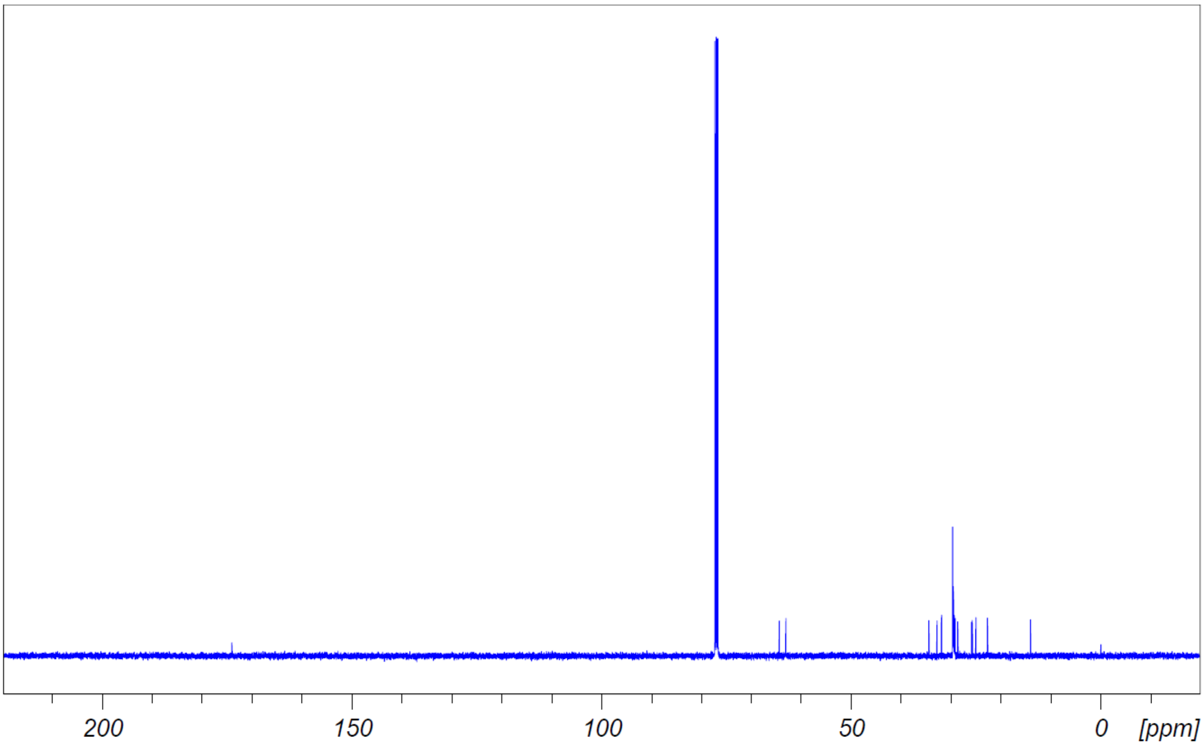

Figure S7. ^{13}C NMR spectrum of **3** (125.68 MHz, 25 °C).

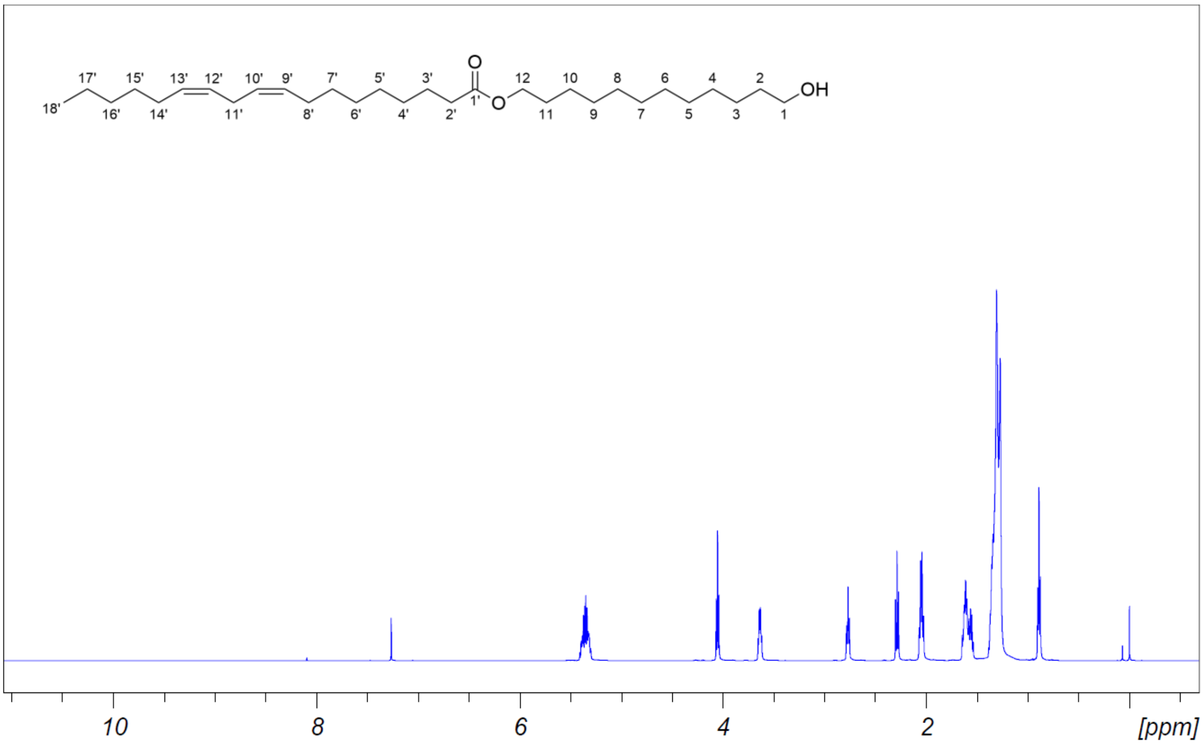

Figure S8. ^{1}H NMR spectrum of **4** (499.82 MHz, 25 °C).

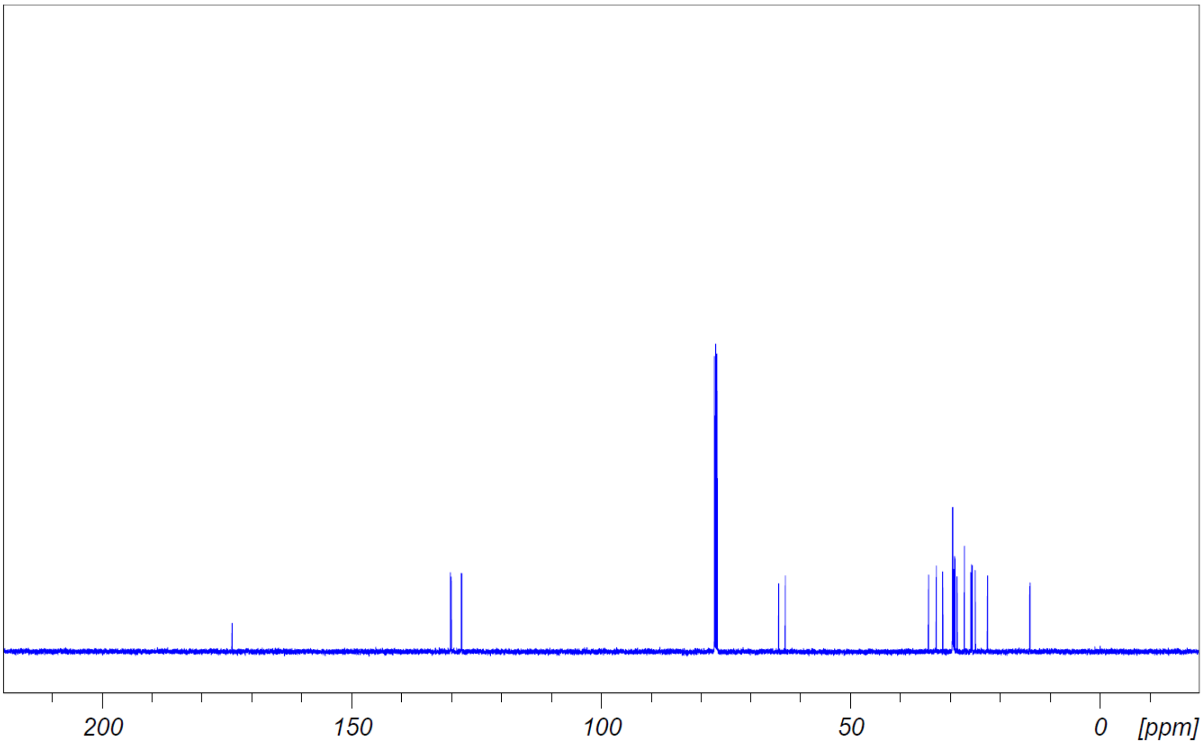

Figure S9. ^{13}C NMR spectrum of **4** (125.68 MHz, 25 °C).

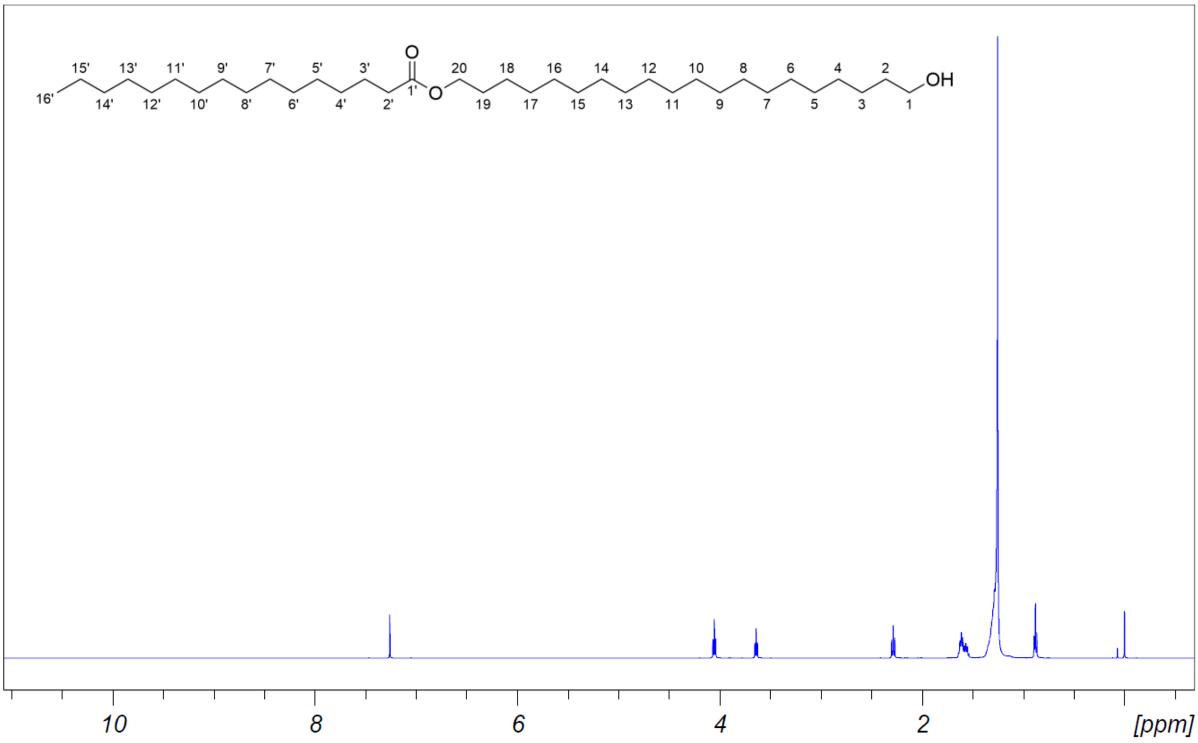

Figure S10. ^{1}H NMR spectrum of **5** (499.82 MHz, 25 °C).

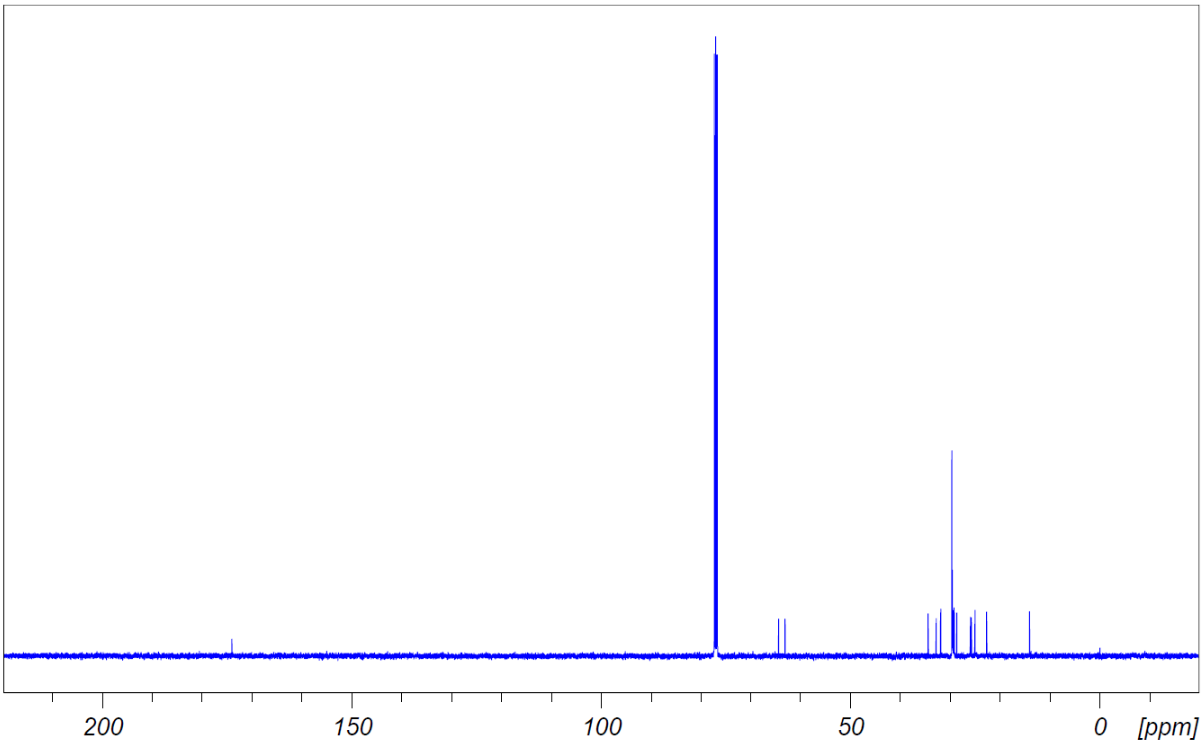

Figure S11. ^{13}C NMR spectrum of **5** (125.68 MHz, 25 °C).

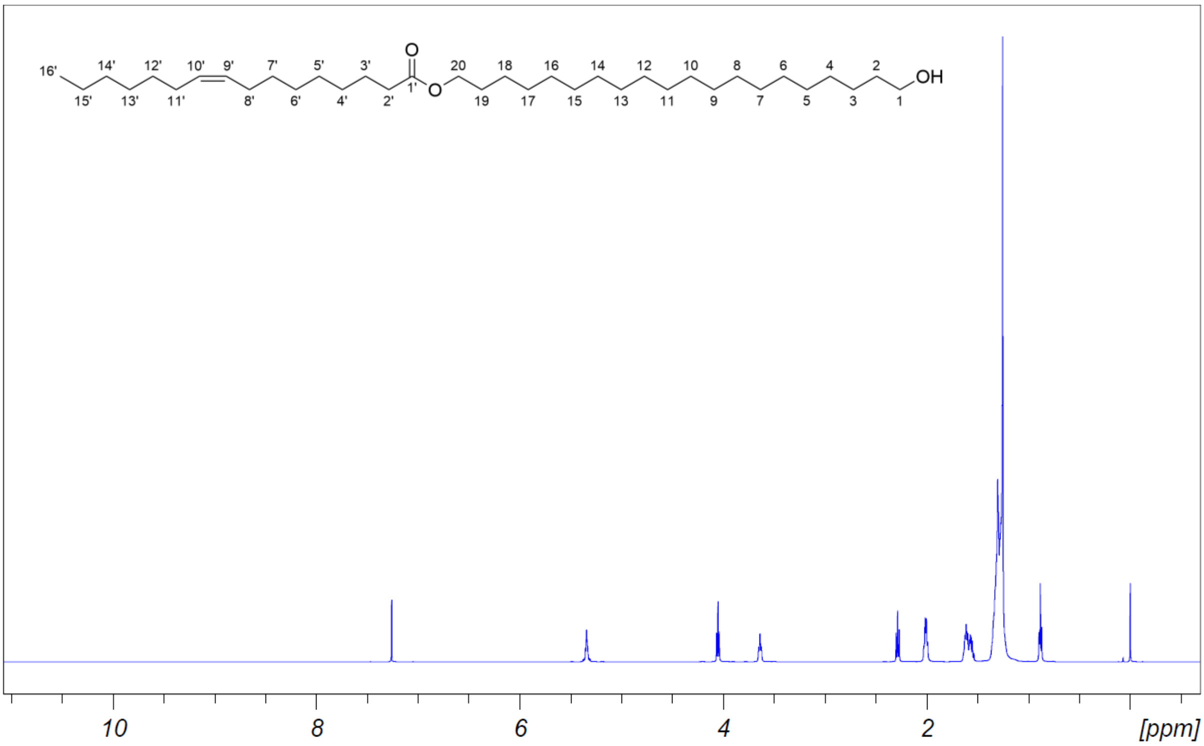

Figure S12. ^{1}H NMR spectrum of **6** (499.82 MHz, 25 °C).

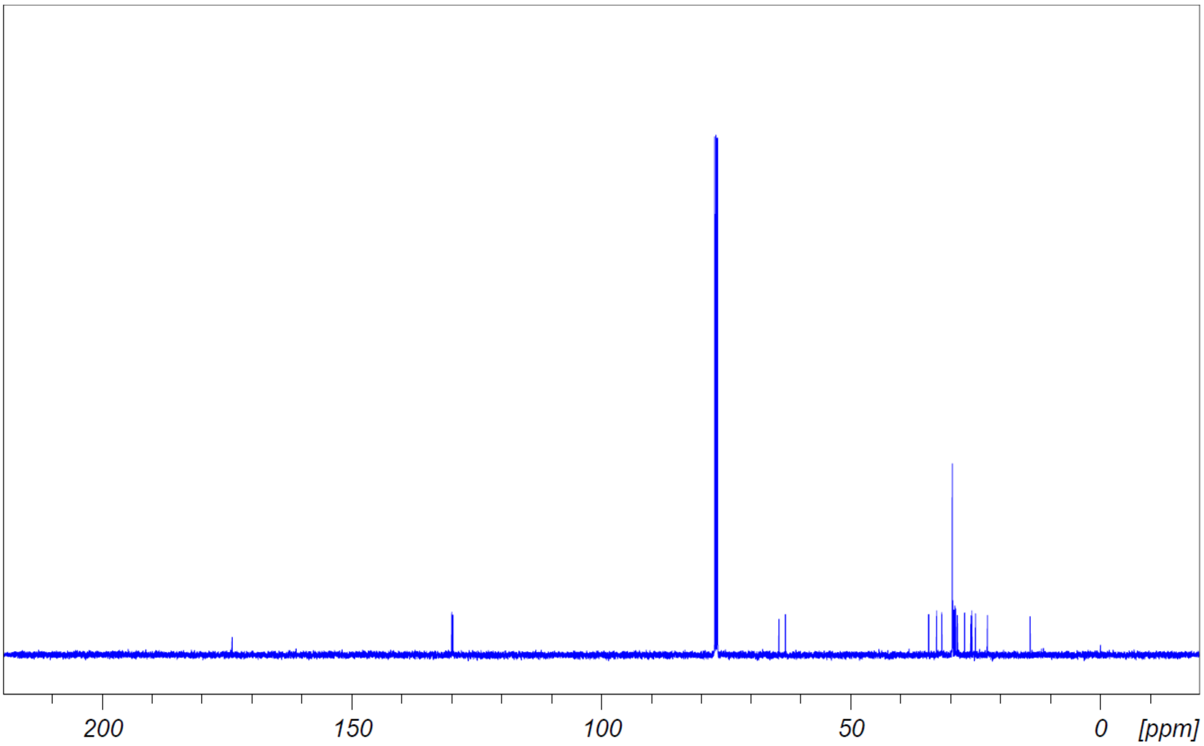

Figure S13. ^{13}C NMR spectrum of **6** (125.68 MHz, 25 °C).

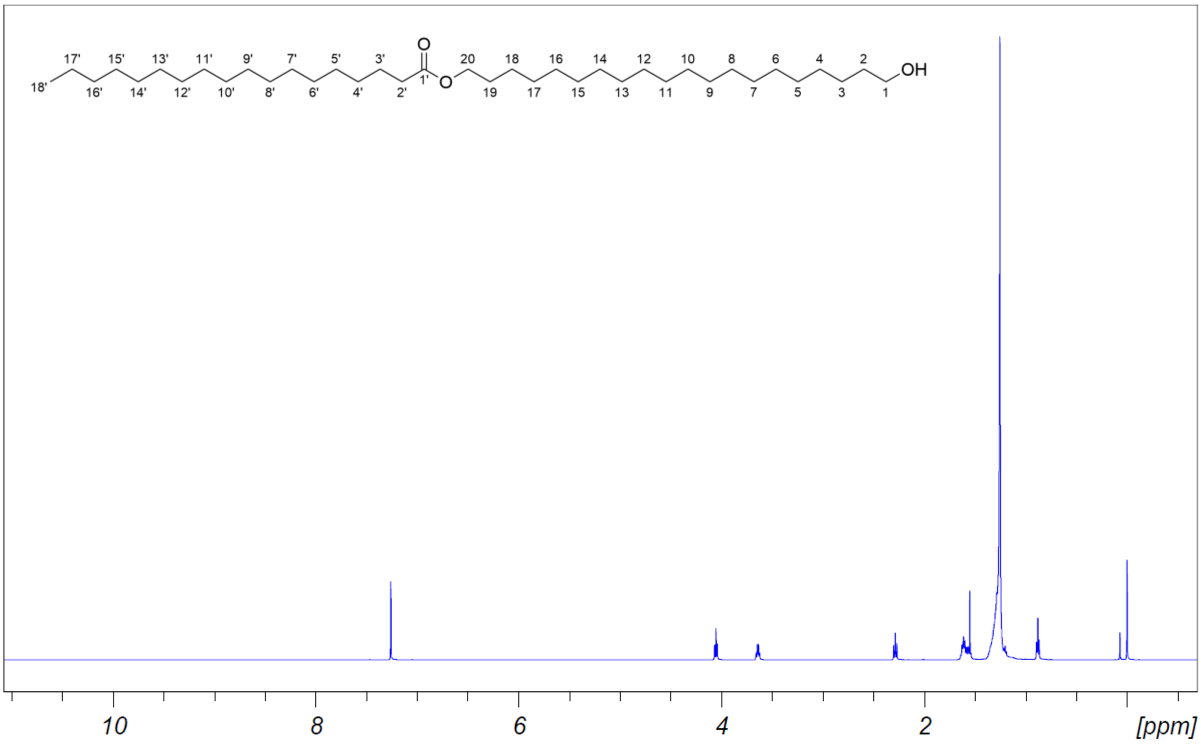

Figure S14. ^{1}H NMR spectrum of **7** (499.82 MHz, 25 °C).

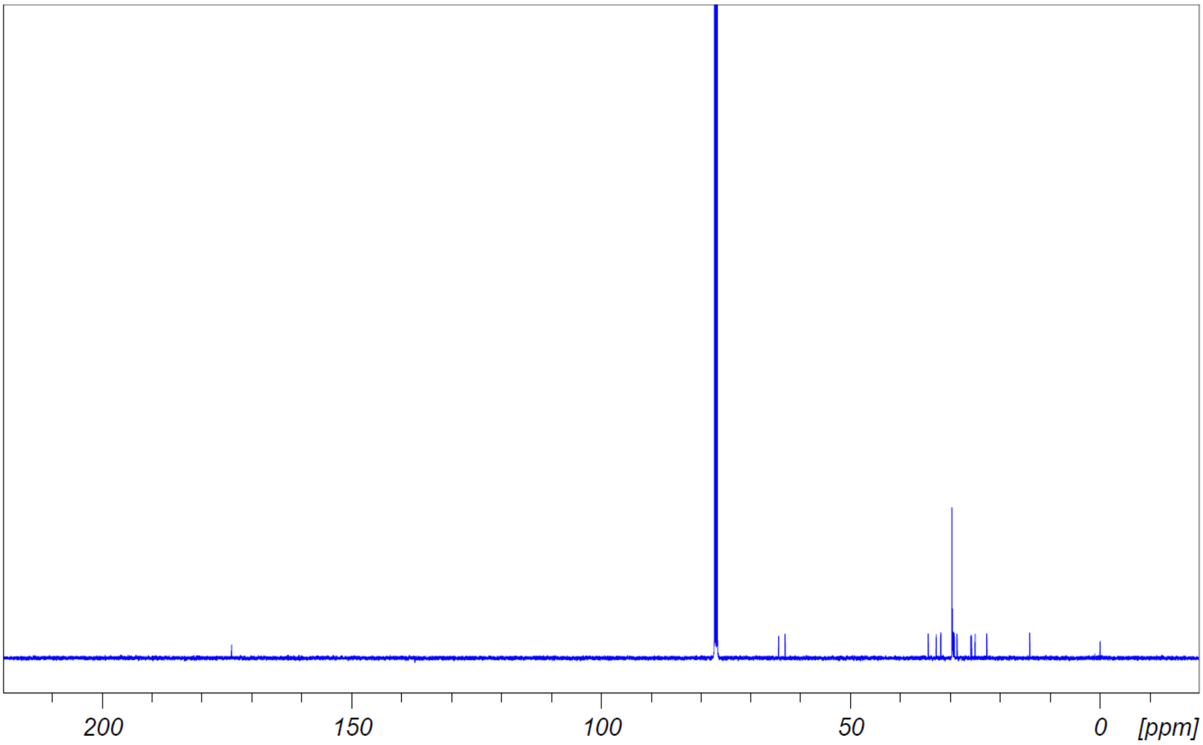

Figure S15. ^{13}C NMR spectrum of **7** (125.68 MHz, 25 °C).

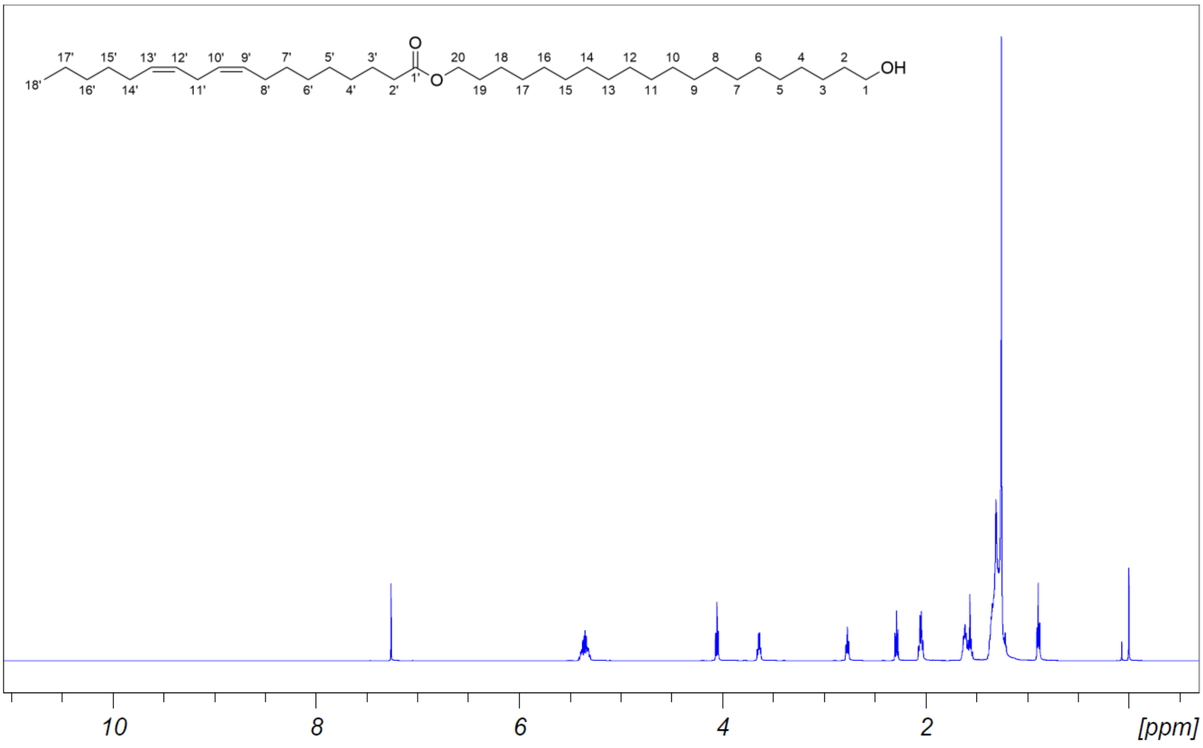

Figure S16. ^{1}H NMR spectrum of **8** (499.82 MHz, 25 °C).

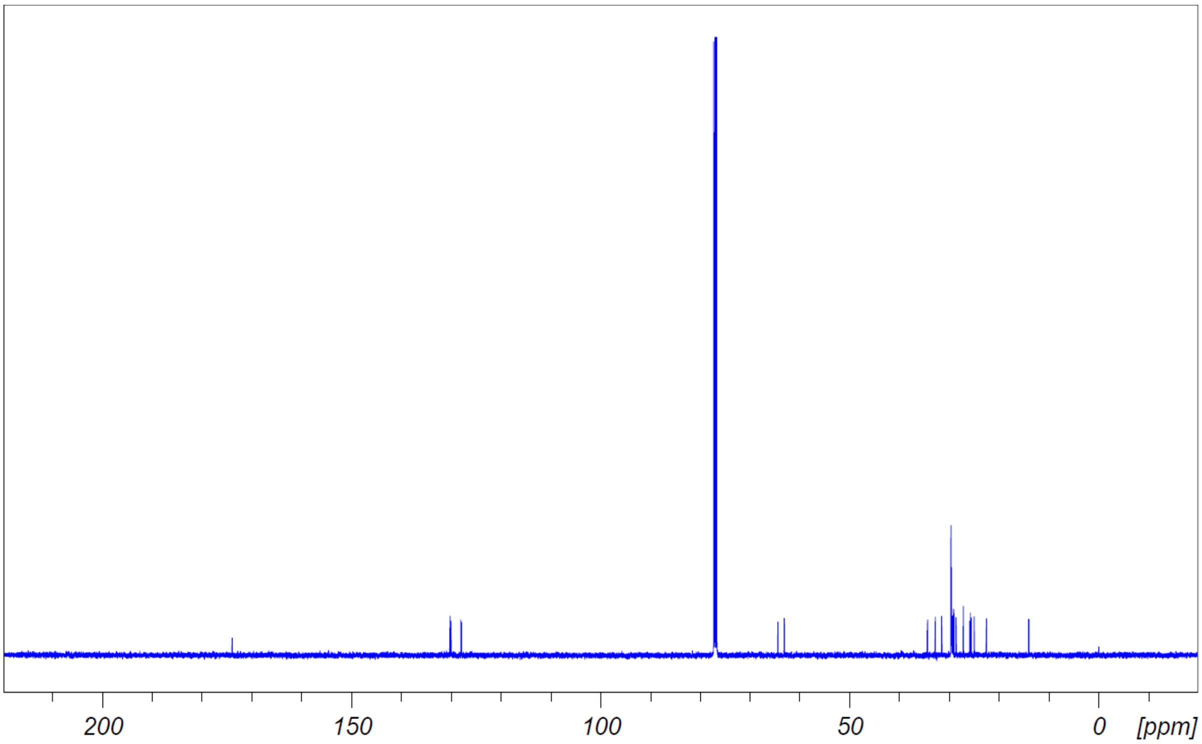

Figure S17. ^{13}C NMR spectrum of **8** (125.68 MHz, 25 °C).

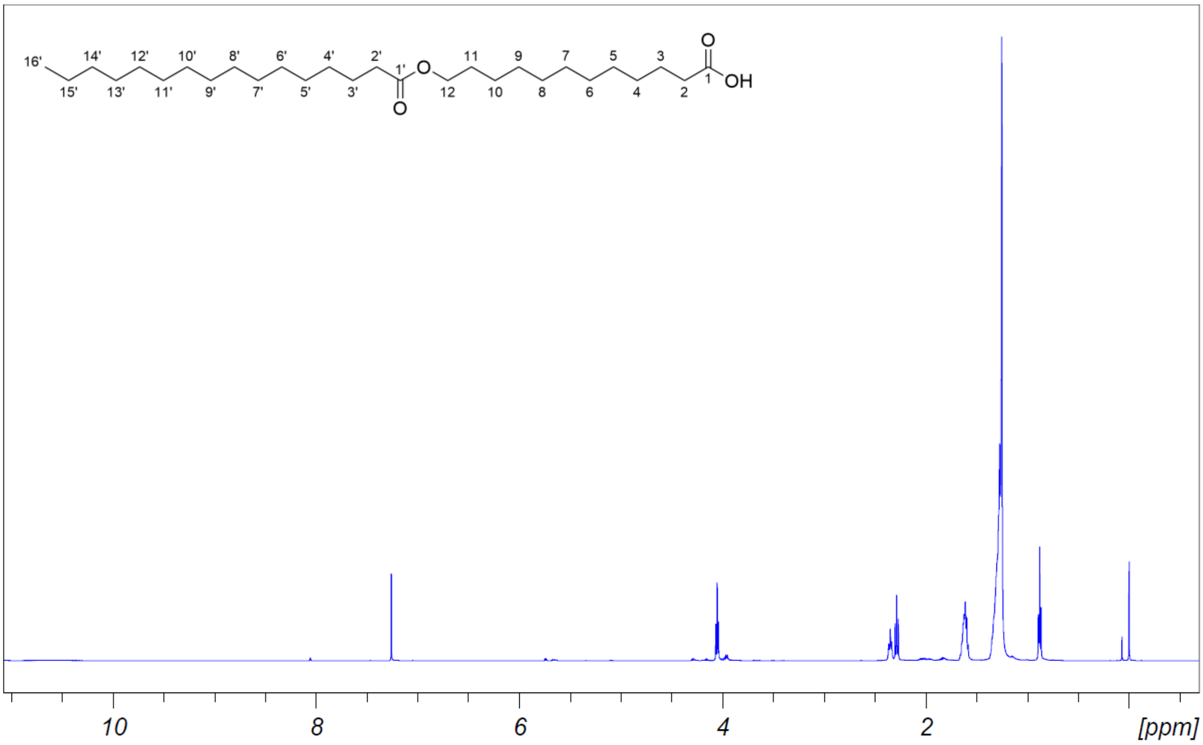

Figure S18. ^{1}H NMR spectrum of **9** (499.82 MHz, 25 °C).

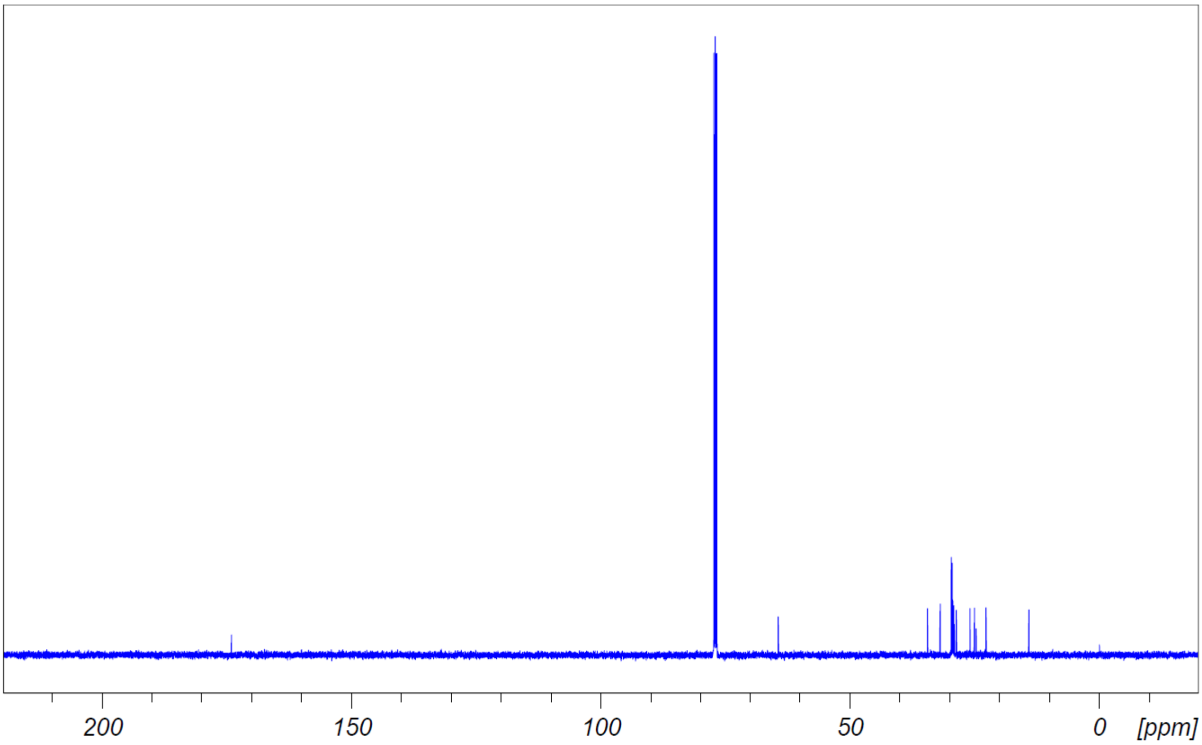

Figure S19. ^{13}C NMR spectrum of **9** (125.68 MHz, 25 °C).

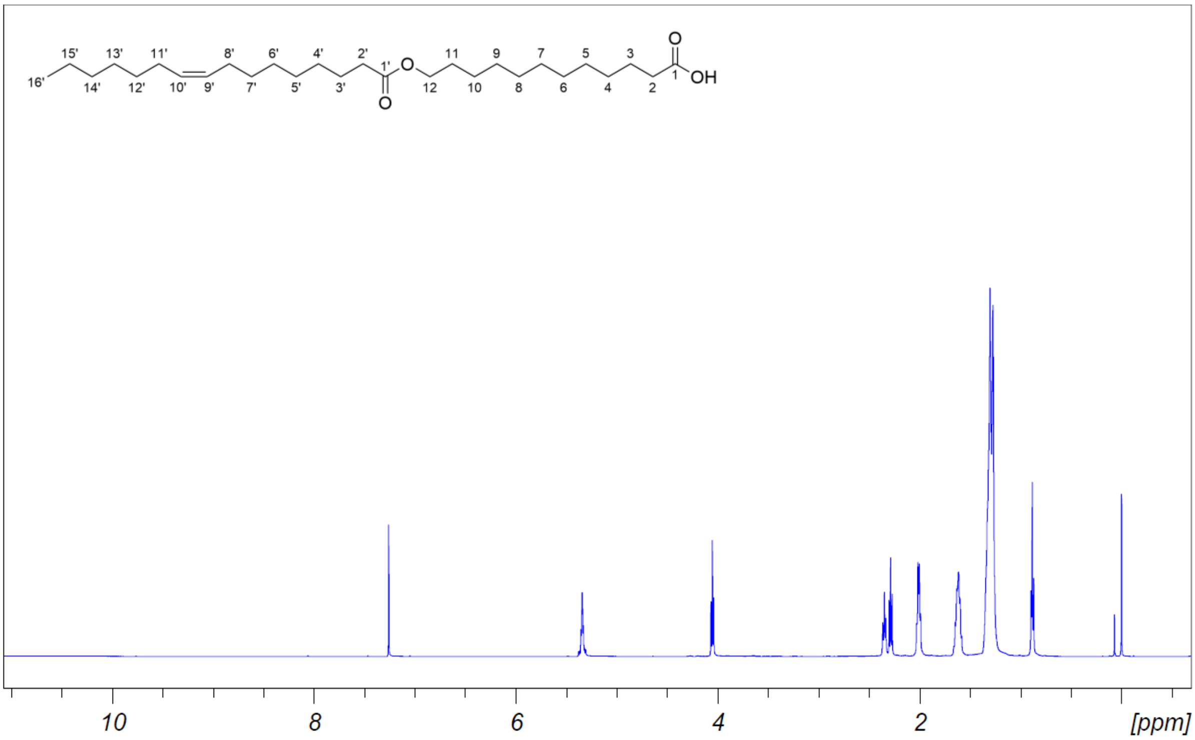

Figure S20. ^{1}H NMR spectrum of **10** (499.82 MHz, 25 °C).

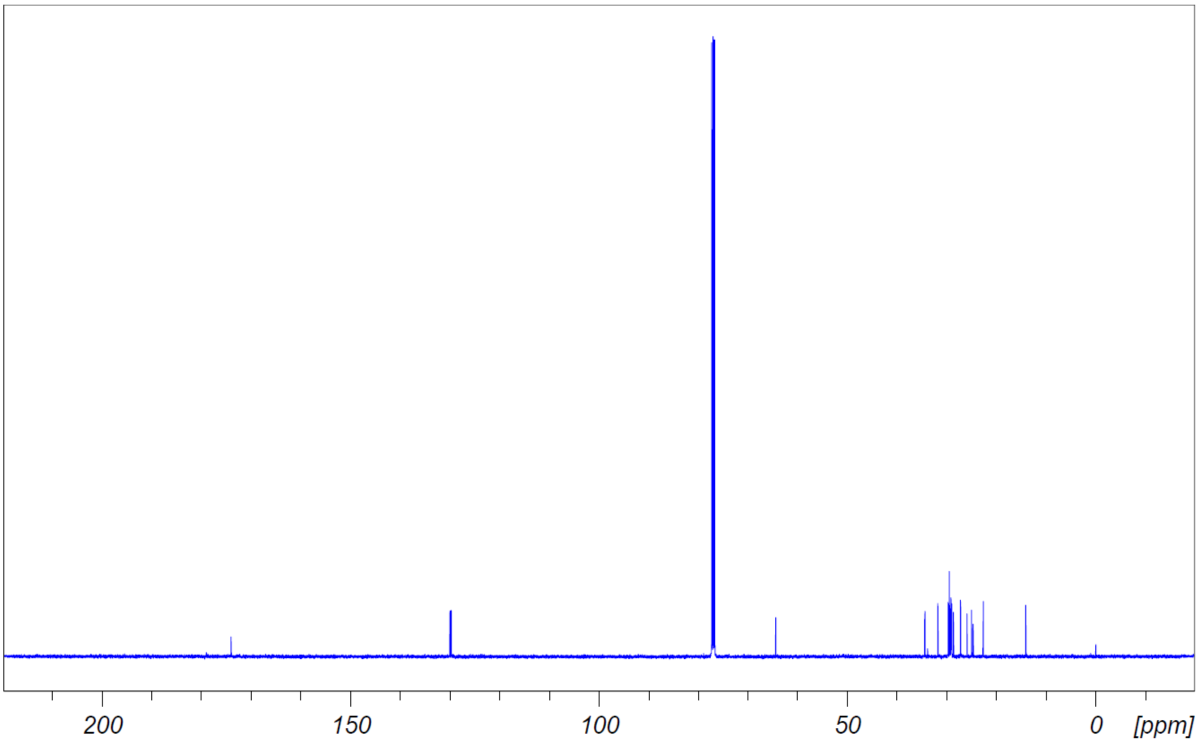

Figure S21. ^{13}C NMR spectrum of **10** (125.68 MHz, 25 °C).

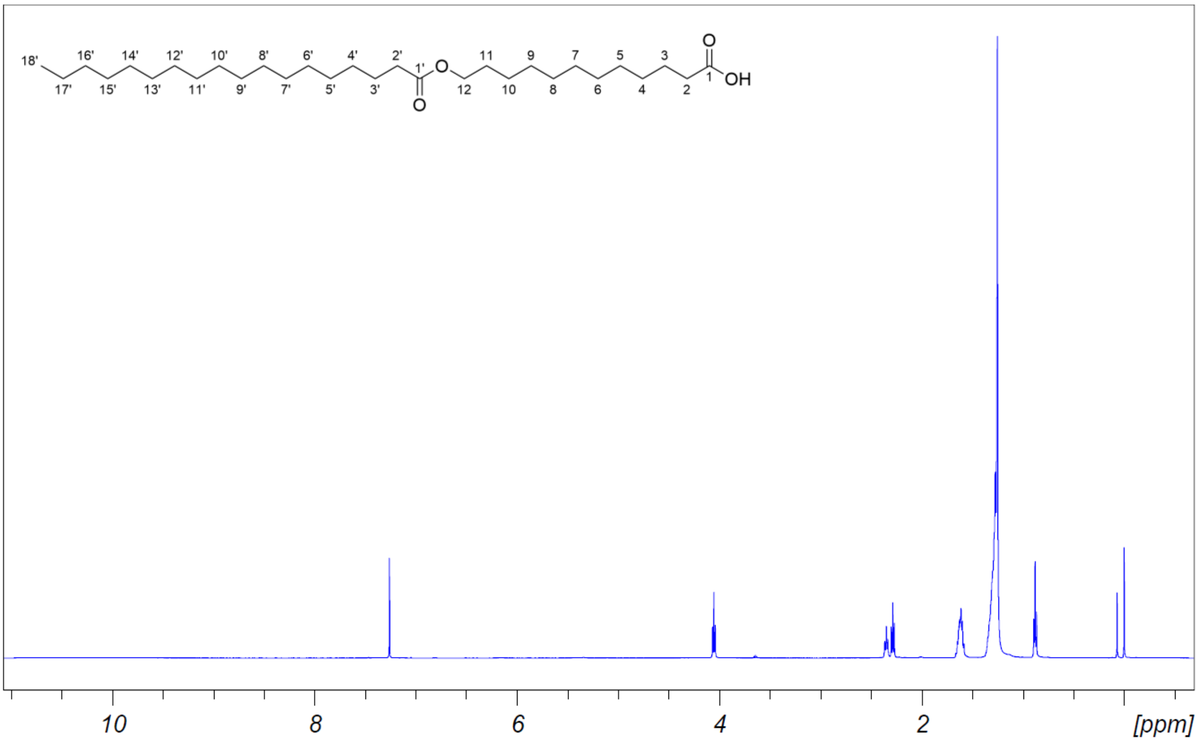

Figure S22. ^{1}H NMR spectrum of **11** (499.82 MHz, 25 °C).

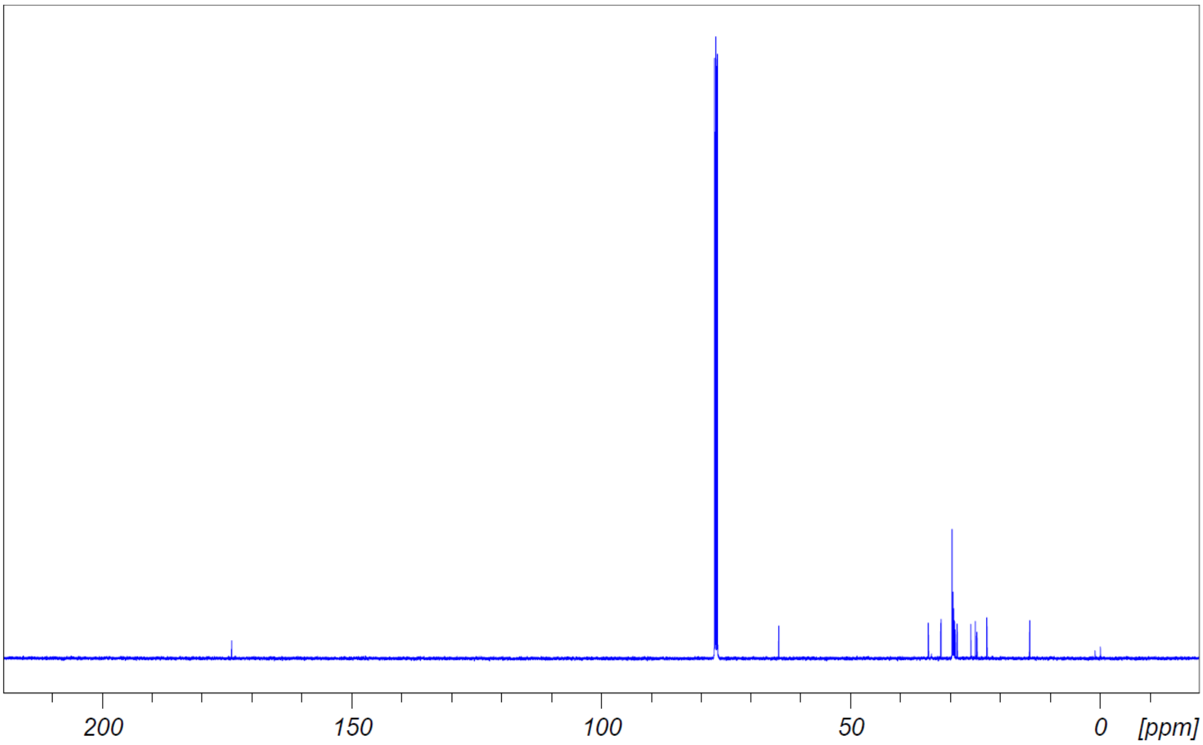

Figure S23. ^{13}C NMR spectrum of **11** (125.68 MHz, 25 °C).

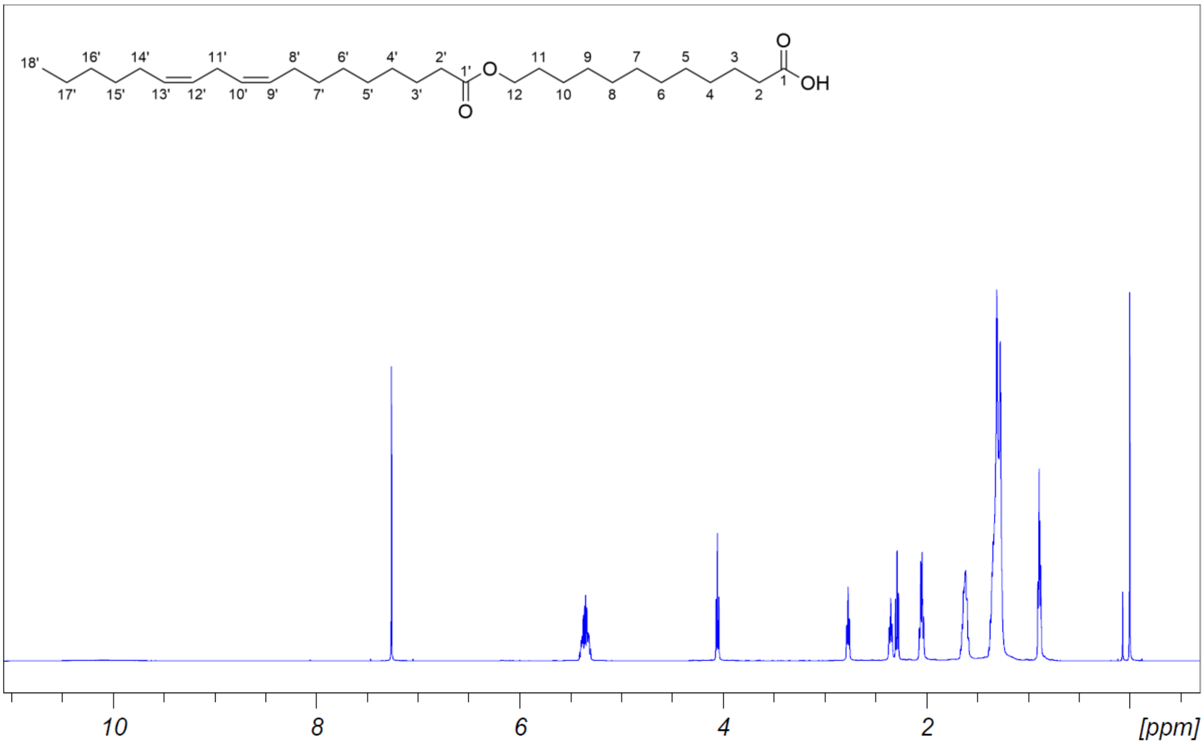

Figure S24. ^{1}H NMR spectrum of **12** (499.82 MHz, 25 °C).

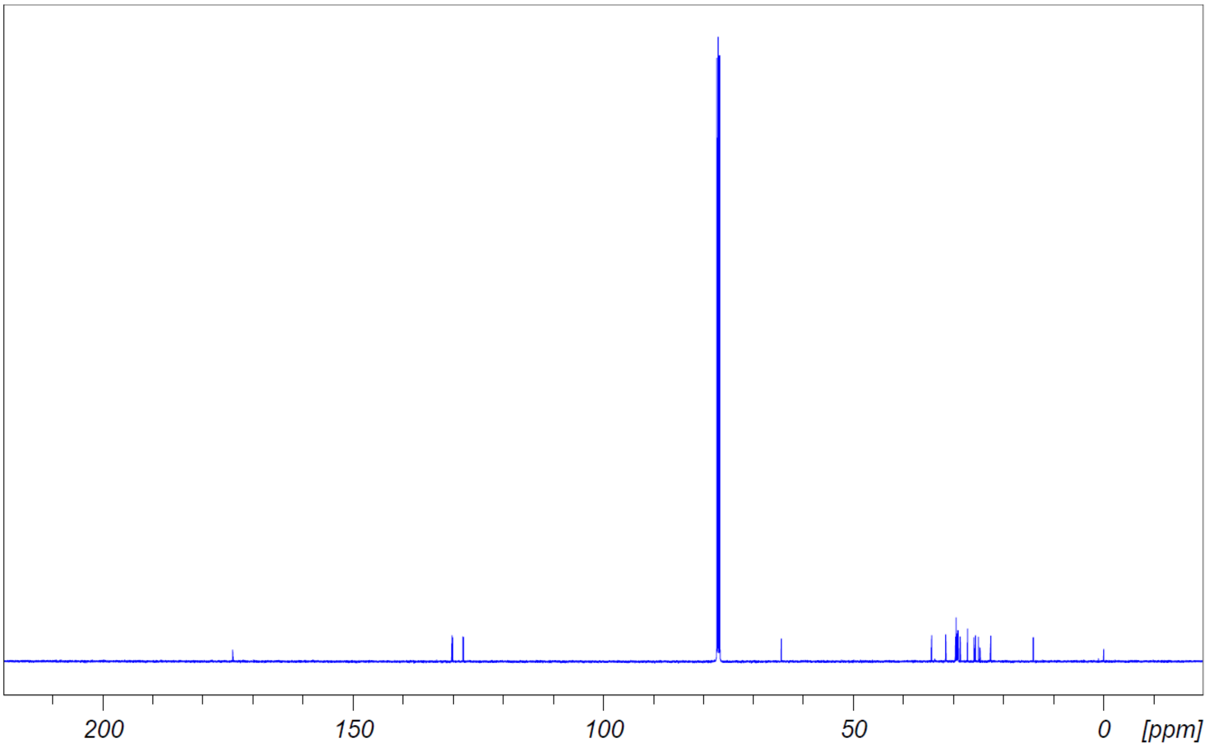

Figure S25. ^{13}C NMR spectrum of **12** (125.68 MHz, 25 °C).

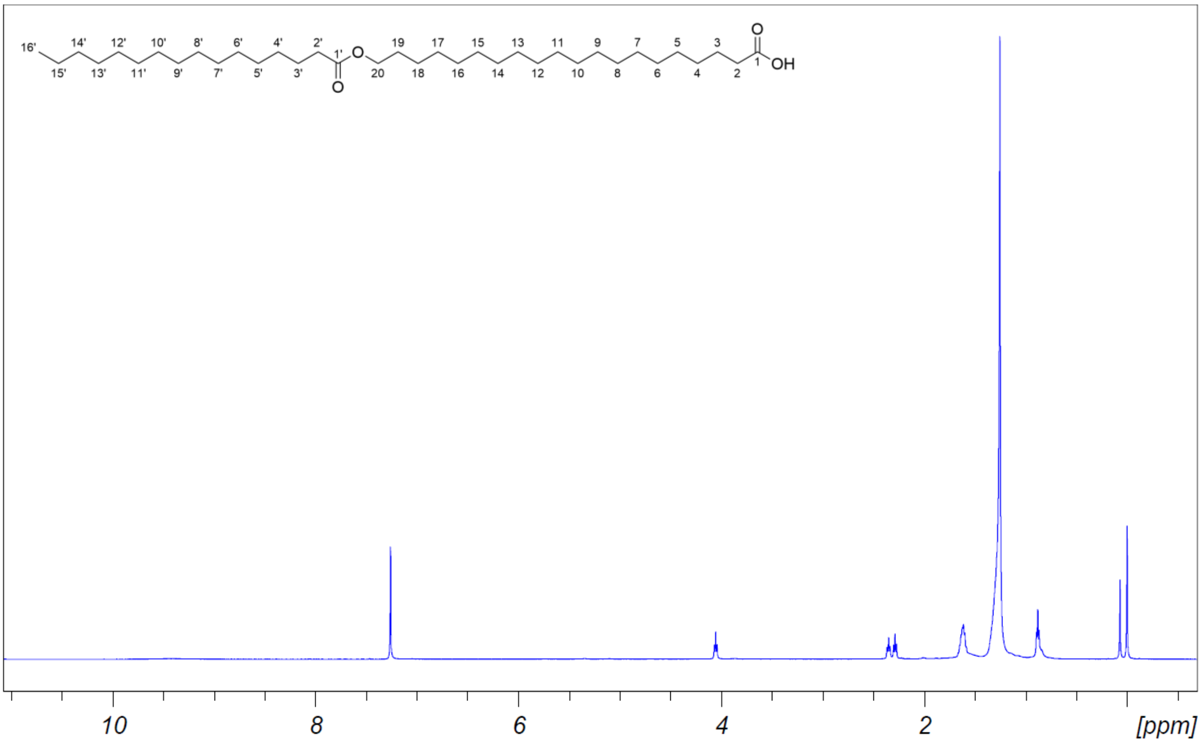

Figure S26. ^{1}H NMR spectrum of **13** (499.82 MHz, 25 °C).

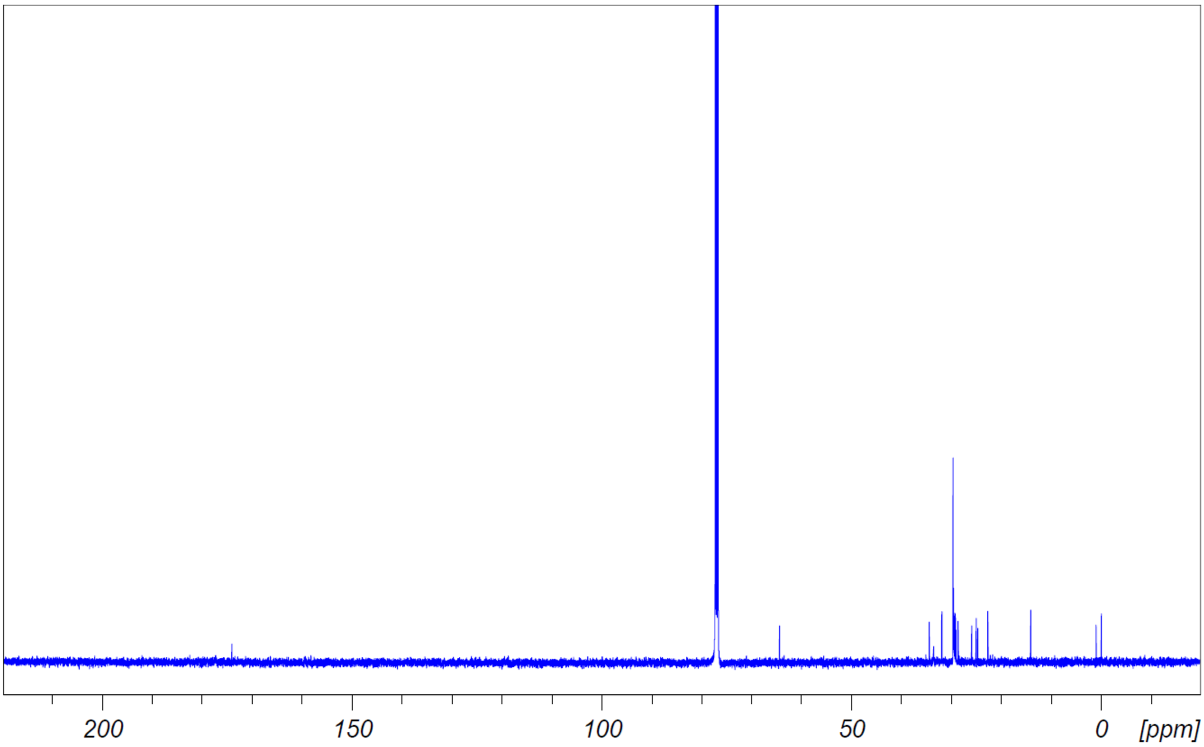

Figure S27. ^{13}C NMR spectrum of **13** (125.68 MHz, 25 °C).

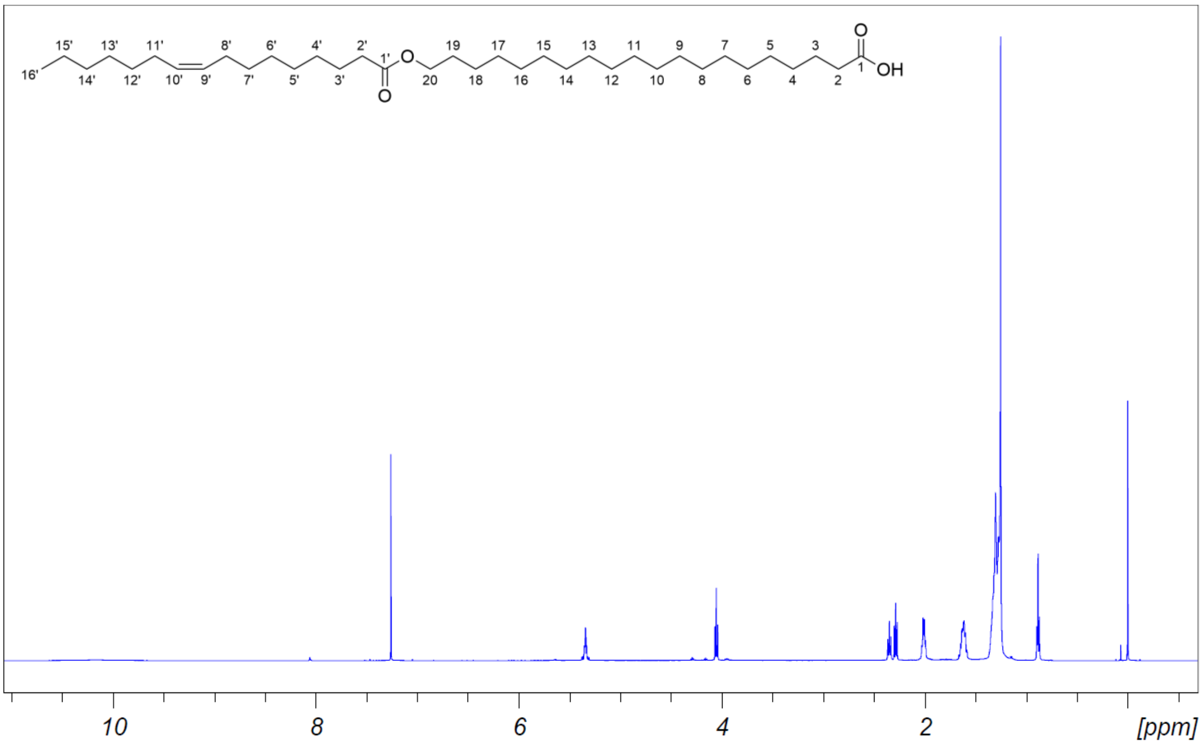

Figure S28. ^{1}H NMR spectrum of **14** (499.82 MHz, 25 °C).

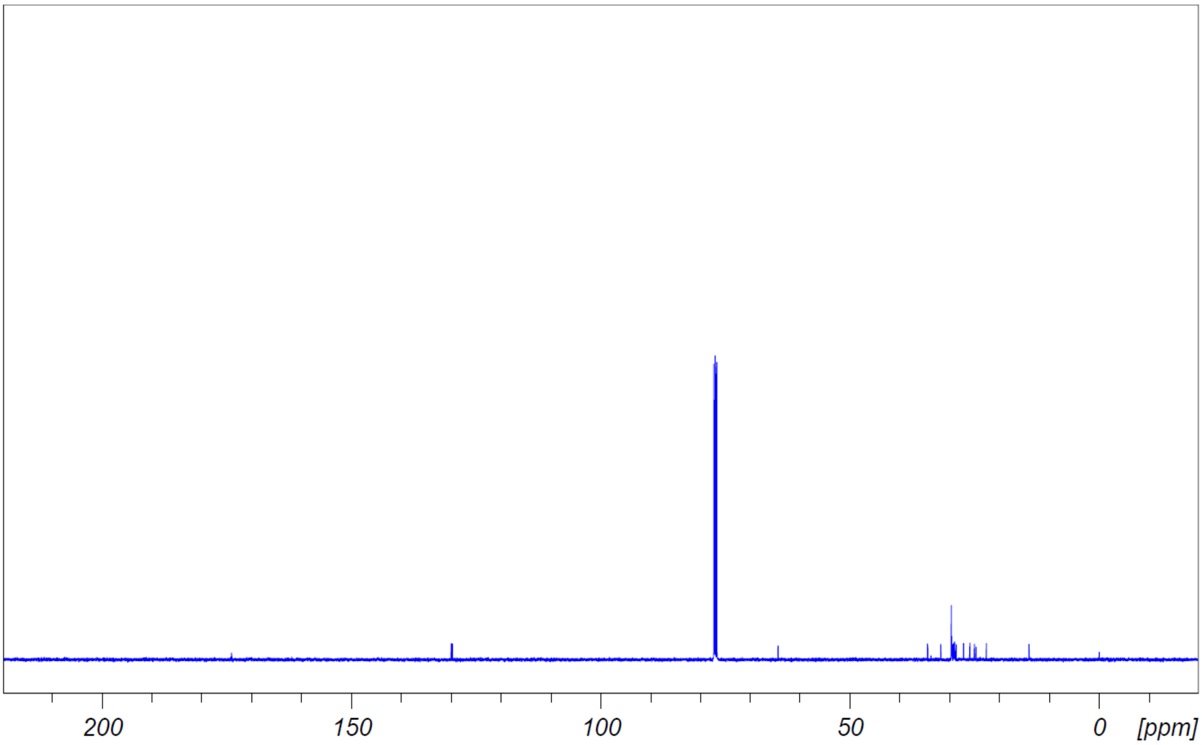

Figure S29. ^{13}C NMR spectrum of **14** (125.68 MHz, 25 °C).

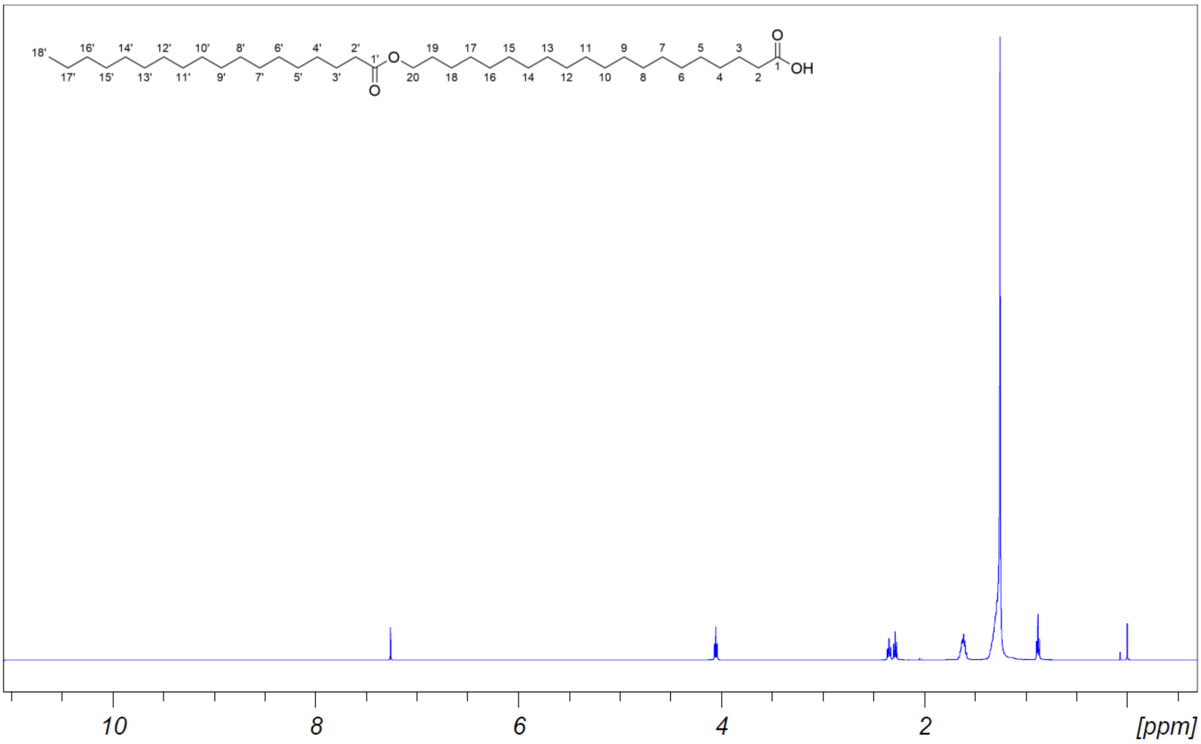

Figure S30. ^{1}H NMR spectrum of **15** (499.82 MHz, 25 °C).

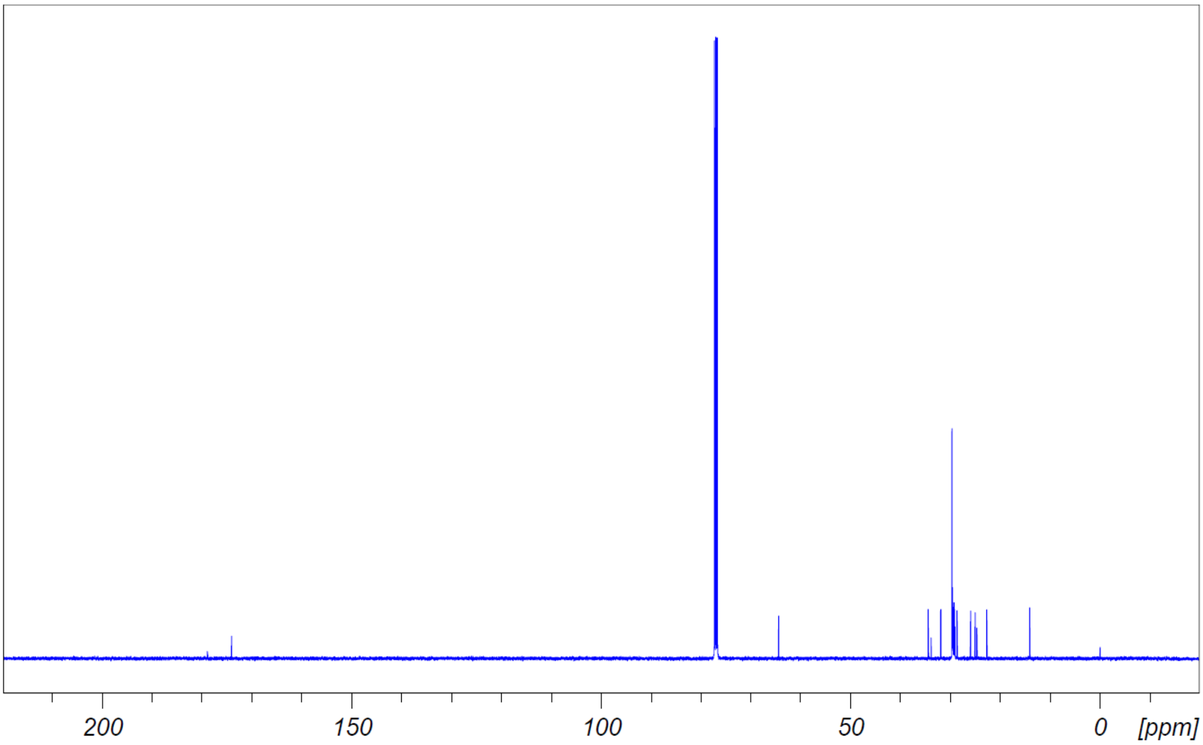

Figure S31. ^{13}C NMR spectrum of **15** (125.68 MHz, 25 °C).

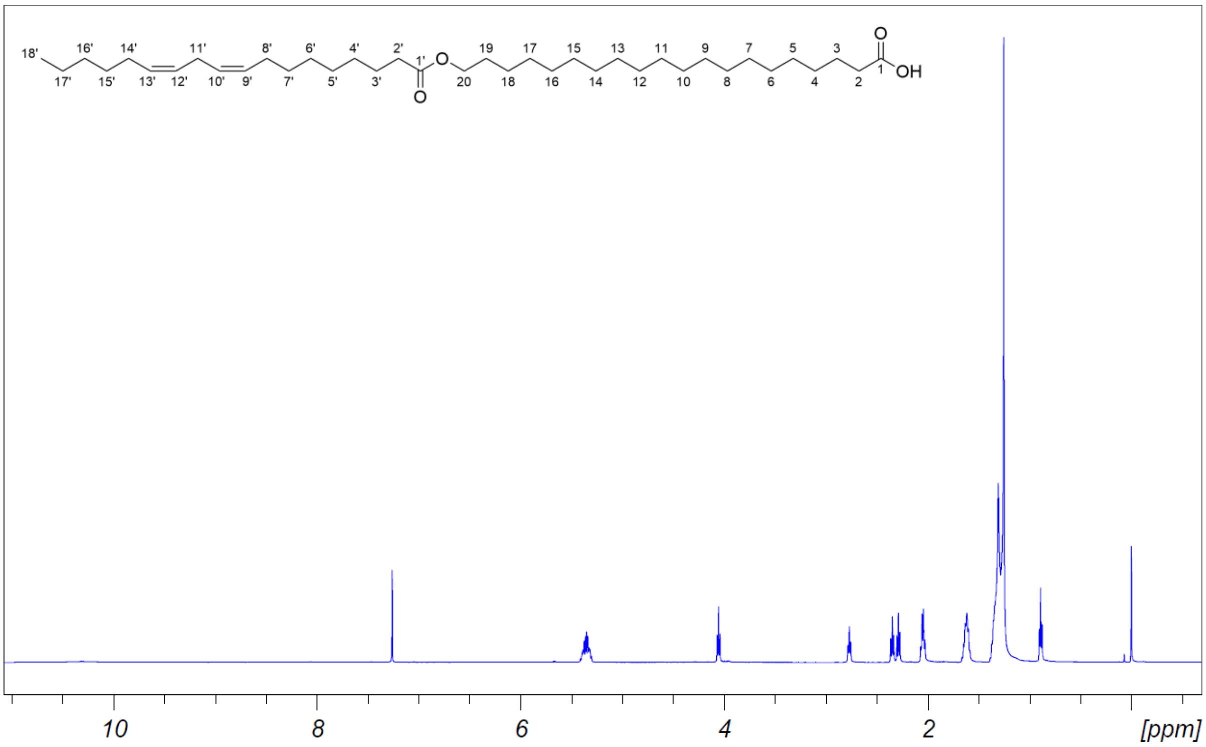

Figure S32. ^{1}H NMR spectrum of **16** (499.82 MHz, 25 °C).

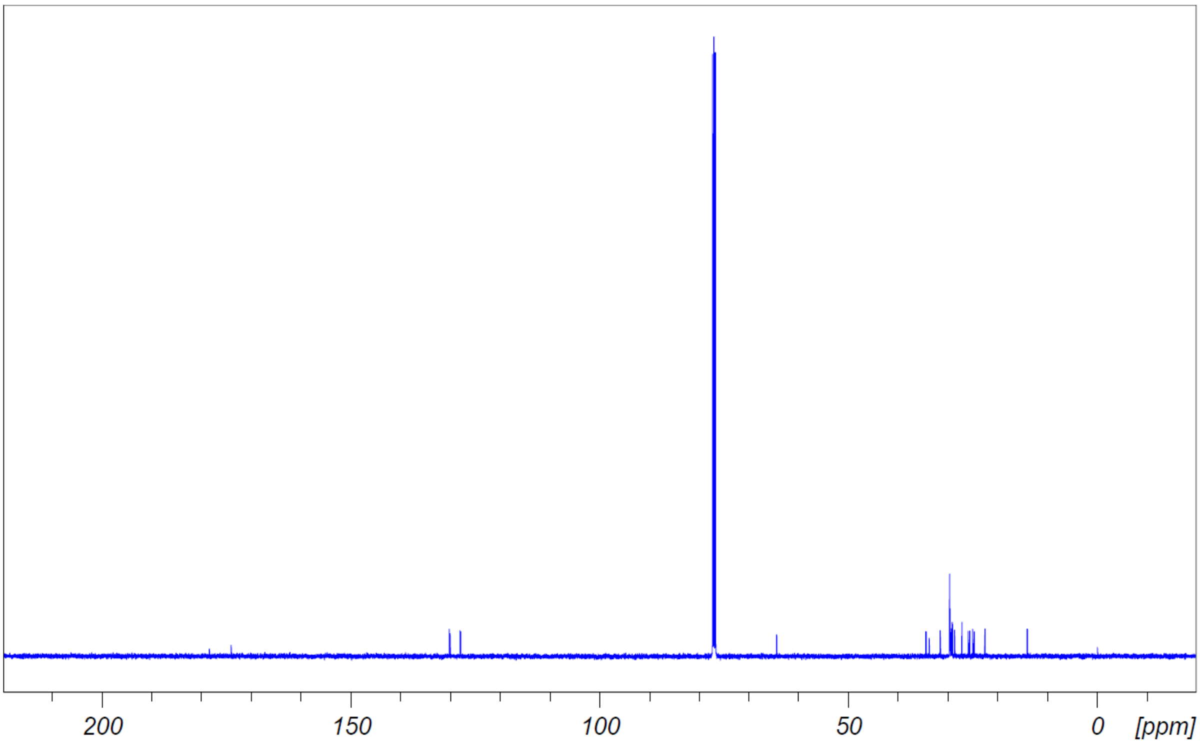

Figure S33. ^{13}C NMR spectrum of **16** (125.68 MHz, 25 °C).
